# Hydrogen Sulfide Inhibits the Development of Atherosclerosis with Suppressing CX3CR1 and CX3CL1 Expression

**DOI:** 10.1371/journal.pone.0041147

**Published:** 2012-07-18

**Authors:** Huili Zhang, Changfa Guo, Duojiao Wu, Alian Zhang, Ting Gu, Liansheng Wang, Changqian Wang

**Affiliations:** 1 Department of Cardiology, Shanghai Ninth People’s Hospital, Shanghai JiaoTong University School of Medicine, Shanghai, China; 2 Department of Cardiac Surgery, Zhongshan Hospital, Fudan University, Shanghai, China; 3 Shanghai Key Laboratory of Organ Transplantation, Zhongshan Hospital, Fudan University, Shanghai, China; 4 Department of Oral and Maxillofacial Pathology, Shanghai Ninth People’s Hospital, Shanghai JiaoTong University School of Medicine, Shanghai, China; 5 Department of Clinical Laboratory, Shanghai Ninth People’s Hospital, Shanghai JiaoTong University School of Medicine, Shanghai, China; Heart Center Munich, Germany

## Abstract

Hydrogen sulfide, as a novel gaseous mediator, has been suggested to play a key role in atherogenesis. However, the precise mechanisms by which H_2_S affects atherosclerosis remain unclear. Therefore, the present study aimed to investigate the potential role of H_2_S in atherosclerosis and the underlying mechanism with respect to chemokines (CCL2, CCL5 and CX3CL1) and chemokine receptors (CCR2, CCR5, and CX3CR1) in macrophages. Mouse macrophage cell line RAW 264.7 or mouse peritoneal macrophages were pre-incubated with saline or NaHS (50 µM, 100 µM, 200 µM), an H_2_S donor, and then stimulated with interferon-γ (IFN-γ) or lipopolysaccharide (LPS). It was found that NaHS dose-dependently inhibited IFN-γ or LPS-induced CX3CR1 and CX3CL1 expression, as well as CX3CR1-mediated chemotaxis in macrophages. Overexpression of cystathionine γ-lyase (CSE), an enzyme that catalyzes H_2_S biosynthesis resulted in a significant reduction in CX3CR1 and CX3CL1 expression as well as CX3CR1-mediated chemotaxis in stimulated macrophages. The inhibitory effect of H_2_S on CX3CR1 and CX3CL1 expression was mediated by modulation of proliferators-activated receptor-γ (PPAR-γ) and NF-κB pathway. Furthermore, male apoE^−/−^ mice were fed a high-fat diet and then randomly given NaHS (1 mg/kg, i.p., daily) or DL-propargylglycine (PAG, 10 mg/kg, i.p., daily). NaHS significantly inhibited aortic CX3CR1 and CX3CL1 expression and impeded aortic plaque development. NaHS had a better anti-atherogenic benefit when it was applied at the early stage of atherosclerosis. However, inhibition of H_2_S formation by PAG increased aortic CX3CR1 and CX3CL1 expression and exacerbated the extent of atherosclerosis. In addition, H_2_S had minimal effect on the expression of CCL2, CCL5, CCR2 and CCR5 in vitro and in vivo. In conclusion, these data indicate that H_2_S hampers the progression of atherosclerosis in fat-fed apoE^−/−^ mice and downregulates CX3CR1 and CX3CL1 expression on macrophages and in lesion plaques.

## Introduction

Hydrogen sulfide is commonly considered to be a toxic gas with the smell of rotten eggs. However, it is generated endogenously during cysteine metabolism in a reaction catalyzed by two pyridoxal phosphate-dependent enzymes, cystathionine β-synthase (CBS, EC4.2.1.22) and cystathionine γ-lyase (CSE, EC4.4.1.1) [1]. CSE is the major H_2_S-producing enzyme in the cardiovascular system, while CBS is the main H_2_S-forming enzyme in the central nervous system. It has become clear that H_2_S fulfills a wide range of physiological functions and plays important roles in the pathogenesis of various cardiovascular diseases, such as hypertension, pulmonary hypertension, and myocardial injury [2]–[3]. Furthermore, recent advances in the understanding of the biological importance of endogenous H_2_S has shed light on the potential role of the gas in atherosclerosis. Wang et al. first reported a direct correlation between endogenous H_2_S and atherosclerosis in apoE^−/−^ mice [4]. Some studies have suggested that H_2_S may hinder the development of atherosclerosis by inhibiting vascular smooth muscle cell proliferation, adhesion molecules expression in endothelial cells and foam cell formation [4]–[6]. However, because of the complexity of the atherogenic process, the anti-atherogenic mechanisms of H_2_S are still far from clear. The present study seeks to investigate whether H_2_S also reduced the expression of chemokines and their receptors, which have been shown to play a key role in inflammatory conditions and atherosclerotic lesion development [7], [8].

In atherogenesis, chemokines and chemokine receptors may coordinate communication between inflammatory cellular components of the peripheral blood and cellular components of the arterial wall, thereby regulating leukocyte influx, capture, efflux and activation, as well as proliferation and/or apoptosis of reside cells in the plaque [7], [8]. The significance of chemokines (CCL2 [monocyte chemotactic protein-1], CCL5 [RANTES] and CX3CL1 [fractalkine]) and their receptors (CCR2, CCR5 and CX3CR1) in atherosclerosis has been demonstrated in animal models and clinical studies [8]–[10]. For instance, CCL2, CCL5, CX3CL1 and CX3CR1 have been identified in human atherosclerotic plaques [10], [11]. Targeting CCR2-CCL2 axis [12], [13], CCR5-CCL5 axis [14]–[16] or CX3CR1-CX3CL1 [17]–[20] axis with genetic and pharmacologic interventions reduced aortic lesion size, decreased macrophage infiltration and increased plaque stability. Because chemokines and chemokine receptors are important in the development of atherosclerosis, identifying the regulation of their expression by H_2_S may contribute to a better understanding of the precise mechanisms by which H_2_S hinders the progression of atherosclerosis.

Accordingly, in the present study, we examined whether H_2_S could regulate the expression of chemokines (CCL2, CCL5, CX3CL1) and chemokine receptors (CCR2, CCR5, CX3CR1) in vivo and in vitro. We found that H_2_S inhibited CX3CR1 expression on stimulated macrophages and reduced aortic CX3CR1 expression in fat-fed apoE^−/−^ mice. The reduction in CX3CR1 expression induced by H_2_S was mediated by peroxisome proliferators-activated receptor-γ pathway. Moreover, H_2_S downregulated CX3CL1 expression in stimulated macrophages and decreased aortic CX3CL1 expression in vivo by suppressing the activation of nuclear factor (NF-κB). However, H_2_S had negligible effect on CCL2-CCR2 axis and CCL5-CCR5 axis in vitro and in vivo. Our findings suggest that H_2_S protects against the formation of atherosclerotic lesions in mice, along with downregulating macrophage CX3CR1 and CX3CL1 expression by a PPAR-γ and NF-κB dependent mechanism.

## Results

### H_2_S inhibits CX3CR1 Expression and CX3CR1-mediated Chemotaxis in IFN-γ or LPS-stimulated Macrophages

**Figure 1:**
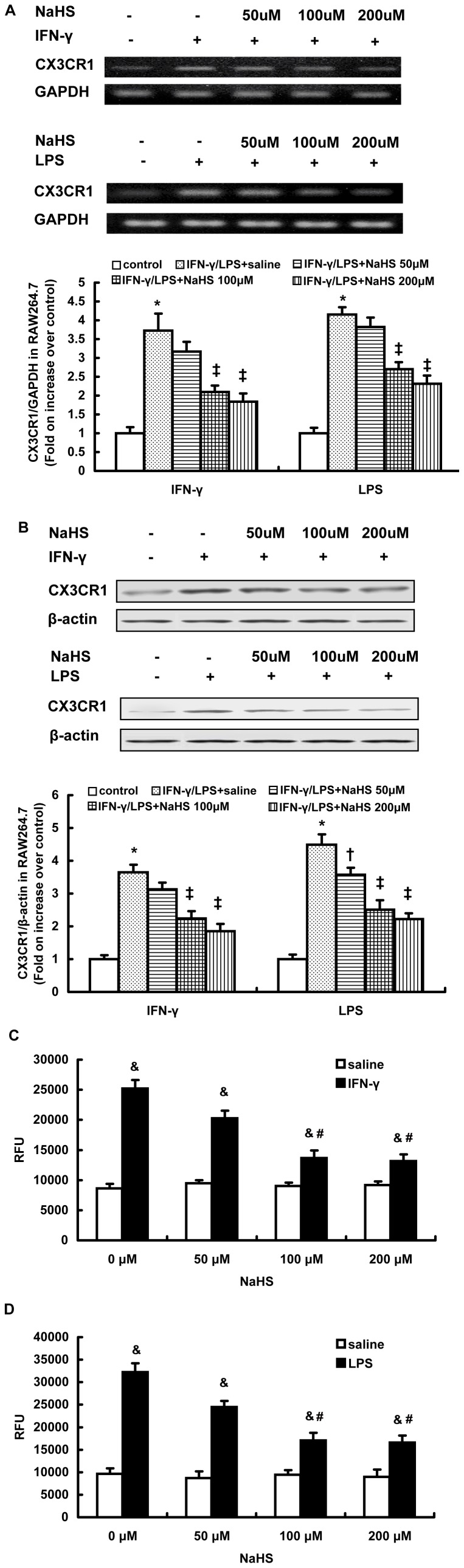
Effect of NaHS on CX3CR1 expression and CX3CR1-mediated chemotaxis in RAW264.7 cells stimulated with IFN-γ or LPS. RT-PCR analysis for CX3CR1 mRNA (A), western blot analysis for CX3CR1 protein expression (B) and chemotaxis towards CX3CL1 (50 ng/ml) (C, D) were carried out as described in Materials and Methods. The data are means ± SEM of at least three independent experiments. *P<0.05, compared with unstimulated cells (control). ‡P<0.05, compared with stimulated cell pretreated with NaHS (50 µM). & P<0.05, compared with unstimulated cells pretreated with NaHS at the same concentration. #P<0.05, compared with stimulated cells pretreated with NaHS at a concentration of 50 µM.

Using RT-PCR and western blot analysis, we found that NaHS dose-dependently decreased the mRNA and protein expression of CX3CR1 in RAW264.7 cells stimulated with either IFN-γ (500 U/ml) or LPS (10 µg/ml) (Figure 1A and 1B). Similar effects of NaHS on CX3CR1 expression were observed in IFN-γ or LPS stimulated mouse peritoneal macrophages (Figure S1). Furthermore, NaHS inhibited the chemotaxis of stimulated RAW264.7 towards soluble CX3CL1 (50 ng/ml) in a dose-dependent manner (Figure 1C and 1D). Treatment with NaHS at a concentration of 100 µM significantly inhibited the migration of stimulated RAW264.7 cells in response to increasing doses of CX3CL1 (Figure S2).

### Suppression of Macrophage CX3CR1 Expression by H_2_S is PPAR-γ Dependent

**Figure 2:**
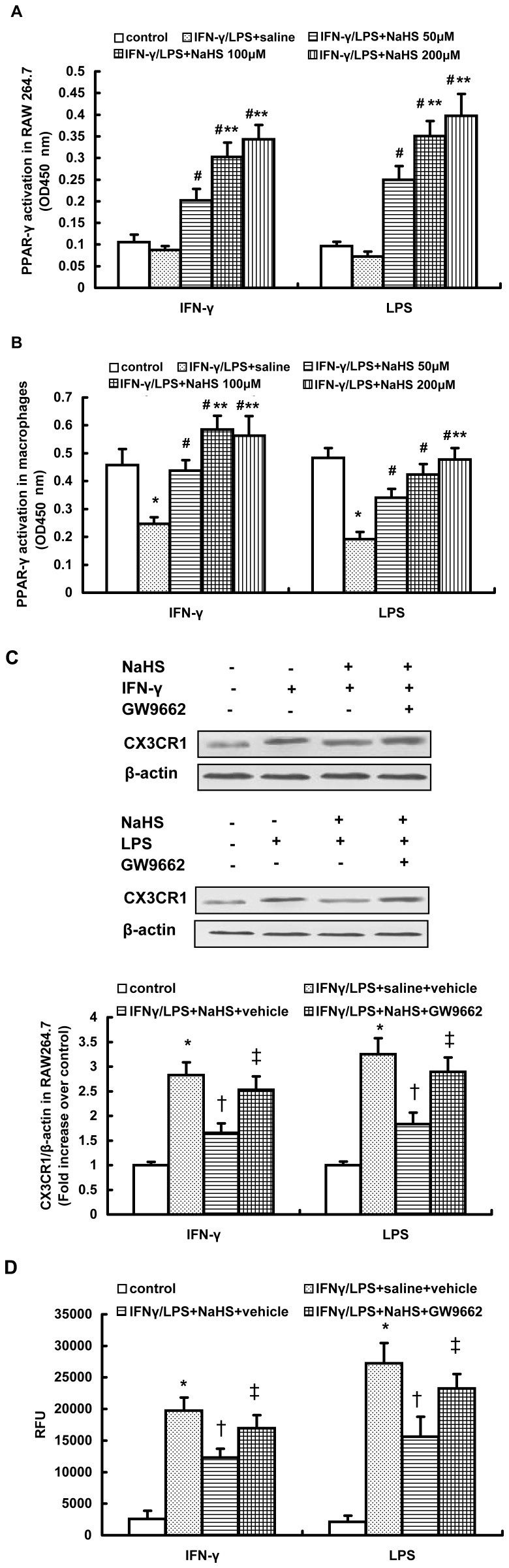
Effect of NaHS on PPAR-γ activation in IFN-γ or LPS-stimulated macrophages. (A, B) RAW264.7 cells (A) or mouse peritoneal macrophages (B) were pre-incubated with saline or NaHS (50 µM, 100 µM, 200 µM) for 6 hours and then stimulated with IFN-γ or LPS for 12 hours in the continuous presence of NaHS or saline. The DNA binding activity of PPAR-γ in nuclear extracts was carried out as described in Materials and Methods. (C, D) RAW264.7 cells were incubated with GW9662 (10 µM) or vehicle for 1 hour, further incubated with NaHS (100 µM) or saline for 6 hours and then stimulated with IFN-γ or LPS for 12 hours in the continuous presence of NaHS or saline. Western blot analysis for CX3CR1 expression (C) and chemotaxis towards CX3CL1 (50 ng/ml) (D) were assayed as described in Materials and Methods. The data are means ± SEM of at least three independent experiments. *P<0.05, compared with unstimulated cells (control). #P<0.05, compared with stimulated cells pretreated with saline. **P<0.05, compared with stimulated cell pretreated with NaHS (50 µM). †P<0.05, compared with stimulated cells treated with saline and vehicle. ‡P<0.05, compared with stimulated cell treated with NaHS and vehicle.

The nuclear receptor PPAR-γ has the inherent capacity to regulate expression of macrophage chemokine receptors (CX3CR1 and CCR2) in lipid-mediated inflammation in atherosclerosis lesions [21], [22]. Therefore, we examined the involvement of PPAR-γ in H_2_S-induced downregulation of CX3CR1 in stimulated macrophages. It was found that unstimulated RAW264.7 cells exhibited a very low level of DNA binding activity for PPAR-γ, whereas a relatively high DNA binding activity for PPAR-γ was observed in mouse peritoneal macrophages (Figure 2A and 2B). This is consistent with a previous report that the basal expression levels of PPAR-γ vary in different macrophages populations [23]. Because the basal expression levels of PPAR-γ differ between RAW264.7 and peritoneal macrophages, the DNA binding activity of PPAR-γ is diverse in these two types of macrophages after exposure to stimulators. Both IFN-γ and LPS resulted in an obvious reduction in the activation of PPAR-γ in mouse peritoneal macrophages, whereas they only slightly decreased the DNA binding activity of PPAR-γ in RAW264.7 cells (Figure 2A and 2B). Regardless of the disparate activation of PPAR-γ between RAW264.7 and peritoneal macrophages after IFN-γ or LPS exposure, NaHS dose-dependently enhanced the DNA binding activity of PPAR-γ in stimulated macrophages (Figure 2A and 2B).

To further test whether PPAR-γ signaling was necessary for the inhibitory effect of H_2_S on CX3CR1 expression in stimulated macrophages, GW9662, a selective PPAR-γ antagonist, was utilized. As shown in Figure 2C, blocking PPAR-γ activity by pretreatment with GW9662 significantly reduced the downregulation of CX3CR1 induced by NaHS in IFN-γ or LPS stimulated RAW264.7 cells. Consequently, NaHS-induced inhibition of CX3CR1-mediated chemotaxis in stimulated RAW264.7 cells was considerably reduced by GW9662 pretreatment (Figure 2D). In addition, GW9662 also abolished NaHS-induced suppression of CX3CR1 expression and CX3CR1-mediated chemotaxis in stimulated peritoneal macrophages (Figure S3). Taken together, these data suggest that H_2_S may inhibit CX3CR1 expression and CX3CR1-mediated chemotaxis in stimulated macrophages in a PPAR-γ-dependent mechanism.

### Overexpression of CSE Inhibits IFN-γ or LPS-induced CX3CR1 Expression by a PPAR-γ Dependent Mechanism

**Figure 3:**
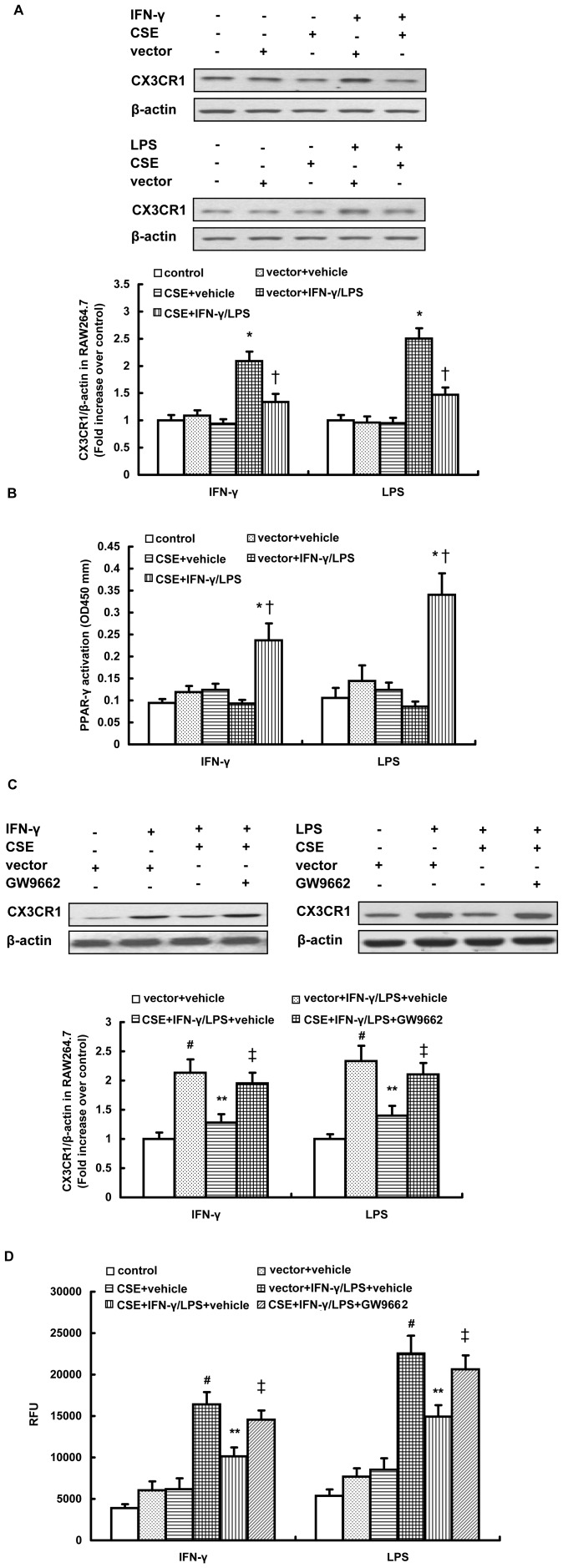
Effect of CSE overexpression on CX3CR1 expression and PPAR-γ activation in stimulated RAW264.7 cells. Cells were transfected with CSE cDNA construct or empty vector and then were stimulated with IFN-γ (500 U/ml) or LPS (10 µg/ml) for 12 hours. Some transfected cells were pre-incubated with GW9962 (10 µM) for 1 hour before addition of IFN-γ or LPS. Western blot analysis for CX3CR1 expression (A, C), PPAR-γ activation (B) and CX3CR1-mediated chemotaxis towards CX3CL1 (50 ng/ml) (D) were assayed as described in Materials and methods. The data are means ± SEM of at least three independent experiments. *P<0.05, compared with unstimulated cells transfected with CSE construct or empty vector. †P<0.05, compared with stimulated cells transfected with empty vector. #P<0.05, compared with mock-transfected and unstimulated cells treated with vehicle. **P<0.05, compared with mock-transfected and stimulated cells treated with vehicle. ‡P<0.05, compared with CSE-transfected and stimulated cells treated with vehicle.

Overexpression of CSE was verified by western blot analysis and an H_2_S synthesis activity assay (Figure S4). As shown in Figure 3A, overexpression of CSE significantly inhibited IFN-γ or LPS-induced CX3CR1 expression in RAW264.7 cells. Furthermore, CSE overexpression significantly enhanced the activation of PPAR-γ in IFN-γ or LPS stimulated RAW264.7 cells (Figure 3B), while mock transfection had a negligible effect on CX3CR1 expression and the nuclear activity of PPAR-γ (Figure 3A and 3B). In agreement with this finding, CSE transfected cells exhibited reduced chemotactic response to CX3CL1 compared with mock-transfected cells after IFN-γ or LPS exposure (Figure 3D). In addition, blockage of PPAR-γ activation by GW9662 distinctly reduced the inhibition of CX3CR1 expression as well as the chemotactic response to CX3CL1 induced by CSE overexpression in stimulated RAW264.7 cells (Figure 3C and 3D). Taken together, these data suggest that CSE overexpression may downregulate CX3CR1 expression and CX3CR1-mediated chemotaxis in stimulated macrophages by activating the PPAR-γ pathway.

### H_2_S Inhibits IFN-γ or LPS-induced CX3CL1 Expression in Stimulated Macrophages by a NF-κB Dependent Mechanism

**Figure 4:**
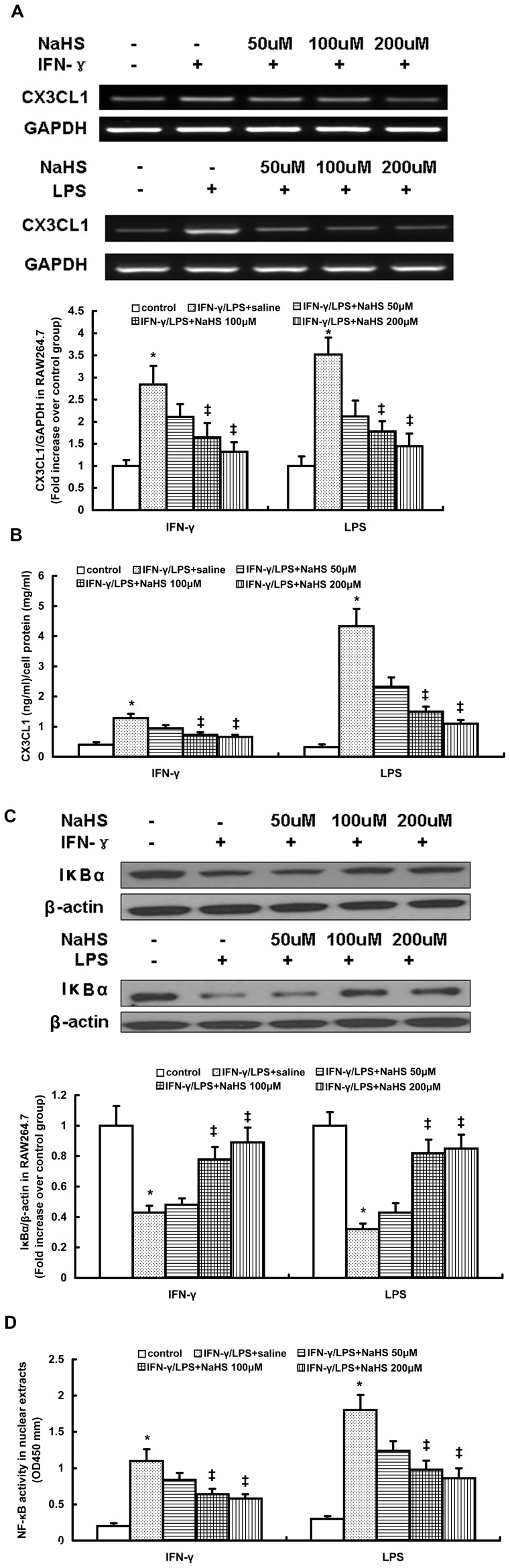
Effect of NaHS on CX3CL1 expression, IκBα content and p65 DNA binding activity in RAW264.7 cells stimulated with IFN-γ or LPS. RT-PCR analysis for CX3CL1 mRNA (A), ELISA for lysate CX3CL1 protein level (B), western blot analysis for IκBα content (C) and ELISA for p65 DNA binding activity in nuclear extracts (D) were carried out as described in Materials and Methods. The data are means ± SEM of at least three independent experiments. *P<0.05, compared with unstimulated cells (control). ‡P<0.05, compared with stimulated cell pretreated with saline.

RAW264.7 cell lysates were analyzed for changes in CX3CL1 mRNA and protein expression (Figure 4A and 4B). Incubation with IFN-γ or LPS significantly increased the concentration of CX3CL1 in RAW264.7 lysates. Preincubation with NaHS decreased lysate CX3CL1 mRNA and protein levels in a concentration dependent manner (Figure 4A and 4B). Stimulation with IFN-γ or LPS also significantly increased the CX3CL1 level in RAW264.7 cell culture media (Table S1). This increase was remarkably reduced by NaHS pretreatment in a dose-dependent manner (Table S1). Similarly, NaHS at a concentration of 100 µM significantly reduced lysate CX3CL1 mRNA and protein levels in mouse peritoneal macrophages stimulate with IFN-γ or LPS (Table S2). It also decreased the CX3CL1 concentration in culture media of stimulated peritoneal macrophages (Table S2). Furthermore, in agreement with these observations, CSE overexpression not only significantly inhibited lysate CX3CL1 mRNA and protein expression but also reduced the CX3CL1 level in culture media of RAW264.7 cells simulated with IFN-γ or LPS (Table S3).

CX3CL1 expression is regulated by the transcriptional activity of NF-κB [24]. Therefore, we examined the involvement of NF-κB in H_2_S-induced downregulation of CX3CL1 in stimulated macrophages. As shown in Figure 4C and Figure 4D, IFN-γ or LPS significantly decreased the IκBα content in RAW264.7 cells and increased the DNA binding activity of NF-κB in nuclear extracts. The nuclear activation of NF-κB induced by IFN-γ or LPS was remarkably suppressed by NaHS in a dose-dependent manner, as characterized by a significant elevation in IκBα content and an obvious reduction in nuclear NF-κB activity (Figure 4C and Figure 4D). Consistent with the findings observed in RAW264.7 cells, NaHS also inhibited nuclear activation of NF-κB in IFN-γ or LPS-stimulated mouse peritoneal macrophages (Figure S5A and Table S2). Moreover, CSE overexpression increased IκBα content and inhibited DNA binding activity of NF-κB in stimulated RAW264.7 cells (Figure S5B and Table S3).

In addition, IFN-γ or LPS significantly upregulated CCR2 and CCR5 mRNA expression and increased lysate CCL2 and CCL5 level in RAW264.7 cells (Figure S6 and Table S4). However, NaHS had negligible effect on CCR2, CCR5, CLL2 and CCL5 expression in stimulated RAW264.7 cells (Figure S6 and Table S4).

### Changes Over Time of Endogenous H_2_S Synthesis During the Development of Atherosclerosis

**Figure 5:**
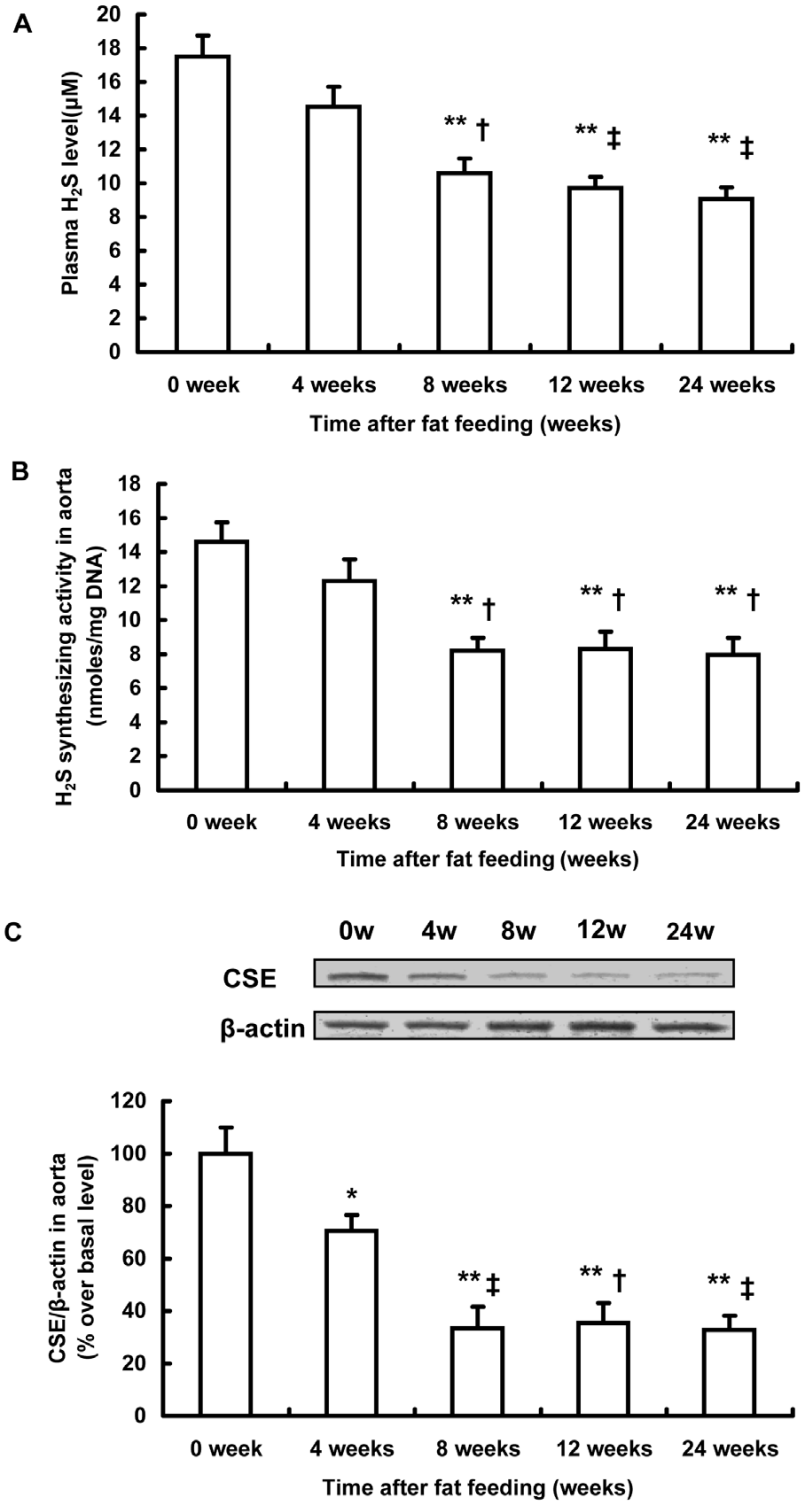
Alterations in H_2_S biosynthesis during the development of atherosclerosis in fat-fed apoE^−/−^ mice. Plasma H_2_S level (A), H_2_S synthesizing activity (B) and CSE expression in aorta (C) were assayed at indicated time points (0, 4, 8, 12, 24 weeks after fat feeding). Results shown are the mean ± SEM (n = 6 animals in each group). *P<0.05, compared with the basal level at 0 weeks. **P<0.01, compared with the basal level at 0 weeks. †P<0.05, compared with mice sacrificed 4 weeks after fat feeding. ‡P<0.01, compared with mice sacrificed 4 weeks after fat feeding.

In an attempt to assess the potential role of H_2_S in atherosclerosis, we observed the time-dependent alterations of H_2_S bio-synthesis in the aorta during the development of atherosclerosis. As shown in Figure 5A, the plasma H_2_S concentration decreased in a time-dependent fashion during the development of atherosclerosis, with a significant reduction after 8-week fat feeding. We also utilized sulfur-sensitive electrode assay to measure the plasma H_2_S level and similar time-dependent changes were obtained (Figure S7). Furthermore, H_2_S synthesizing activity and CSE expression in the aorta gradually reduced in a time-dependent manner (Figure 5B and 5C). These data indicate that a deficiency of endogenous H_2_S formation in the aorta accompanied the development and progression of atherosclerosis. The insufficiency of endogenous H_2_S in atherosclerosis may have been due to the downregulation of CSE expression and reduced CSE activity in the aorta.

### Effect of H_2_S on the Development of Atherosclerosis

**Figure 6:**
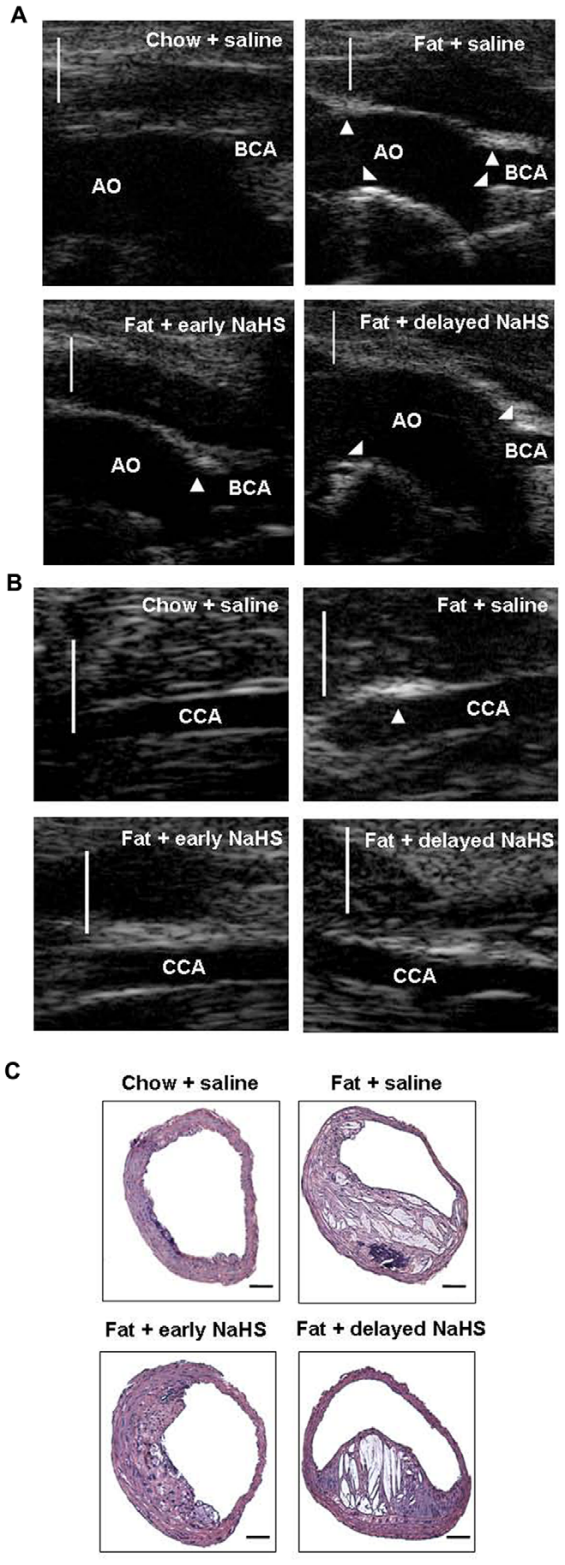
Effect of NaHS on the development of atherosclerosis in fat-fed apoE^−/−^ mice. (A) Representative UBM images were taken from the aortic arch (AO) and BCA in mice with chow and saline, mice with fat feeding and saline, mice with fat feeding and early NaHS treatment or mice with fat feeding and delayed NaHS treatment. (B) Representative UBM images were taken from CCA in mice with chow and saline, mice with fat feeding and saline, mice with fat feeding and early NaHS treatment or mice with fat feeding and delayed NaHS treatment. Scale bar for UBM  =  1 mm. Atherosclerotic plaques are identified by arrows. (C) Representative H&E staining images were taken from BCA in mice with chow and saline, mice with fat feeding and saline, mice with fat feeding and early NaHS treatment or mice with fat feeding and delayed NaHS treatment. Scale bar for histological images  =  100 µm.

NaHS, an H_2_S donor, was given to mice during the early stage of atherosclerosis (4 weeks after fat feeding, early NaHS treatment). 24 weeks after fat feeding, mice were sacrificed and the extent of atherosclerotic lesions was assessed by ultrasound biomicroscopy (UBM) and histological analysis. UBM assessment showed that early NaHS treatment significantly impeded plaque development in the aorta, as characterized by a significant decrease in the brachiocephalic artery (BCA) plaque size, common carotid artery (CCA) intima-media complex thickness (IMT) and the aortic arch IMT (Figure 6A, Figure 6B and table 1). It was also verified by histological analysis that early NaHS treatment significantly reduced the plaque area and lipid core in BCA (Figure 6C and table 2). On the other hand, we delayed the commencement of NaHS treatment until the plaques were fully established as identified by UBM (12 weeks after fat feeding, delayed NaHS treatment). We found that delayed NaHS treatment significantly inhibited the progression of atherosclerosis (Figure 6, Table 1 and Table 2). However, when compared to delayed NaHS treatment, early NaHS treatment induced a greater reduction in plaque size and IMT. All of these findings suggest that H_2_S may have an anti-atherogenic benefit and that this effect is more potent when applied during the early stage of atherosclerosis.

**Table 1:** Effect of treatment with NaHS on plaque development in aortic arch, brachiocephalic artery (BCA) and common carotid artery (CCA) by ultrasound biomicroscopy (UBM) technology.

	Aortic arch IMT	CCA IMT	Plaque area in BCA
	(mm)	(mm)	(mm^2^)
chow + saline	0.0537±0.0069	0.0415±0.0051	0.0511±0.0064
Fat + saline	0.1450±0.0076*	0.1213±0.0115*	0.1660±0.0139*
Fat + early NaHS treatment	0.0879±0.0065**	0.0621±0.0062** #	0.0816±0.0137** #
Fat + delayed NaHS treatment	0.0969±0.0074**	0.0930±0.0044**	0.1226±0.0064**

* P<0.05, vs. chow + saline group;

** P<0.05, vs. fat + saline group;

# P<0.05, vs. fat + delayed NaHS treatment group.

**Table 2:** Effect of treatment with NaHS on plaque development in brachiocephalic artery (BCA) by histology assessment.

	Plaque area (mm^2^)	Lipid core (%)
chow + saline	0.0358±0.0051	9.53±1.28
Fat + saline	0.1480±0.0152*	39.63±4.25*
Fat + early NaHS treatment	0.0641±0.0107** #	15.81±1.87** #
Fat + delayed NaHS treatment	0.1013±0.0052**	25.72±1.96**

* P<0.05, vs. chow + saline group;

** P<0.05, vs. fat + saline group;

# P<0.05, vs. fat + delayed NaHS treatment group.

**Figure 7:**
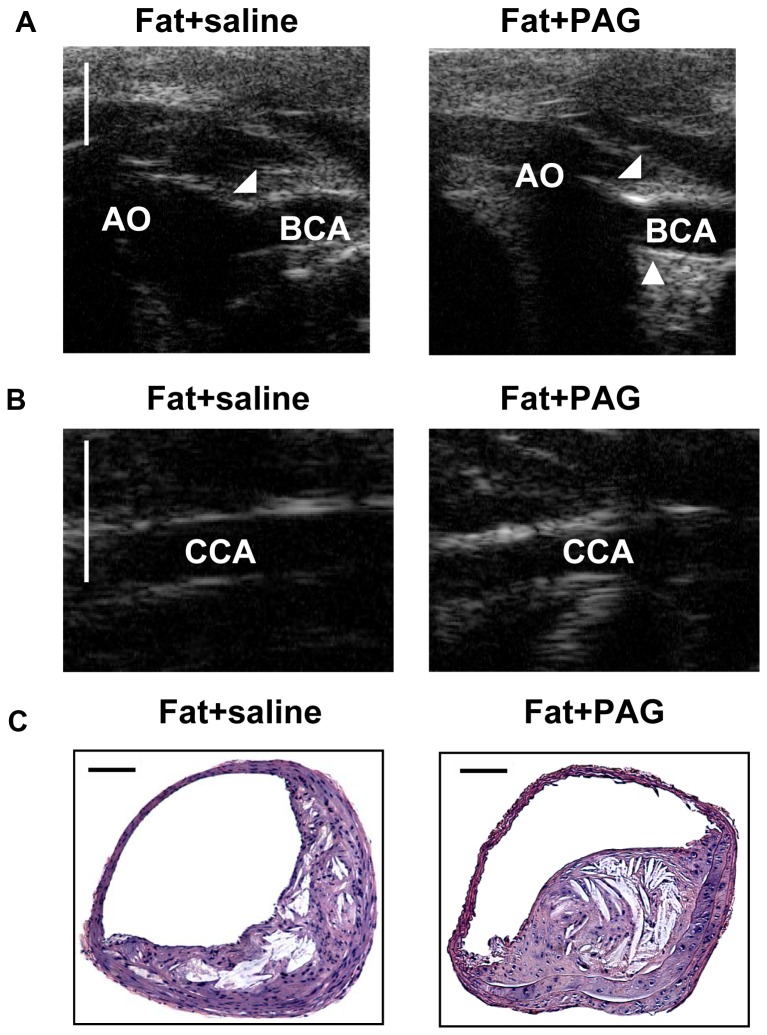
Effect of PAG on the development of atherosclerosis in fat-fed apoE^−/−^ mice. (A) Representative UBM images were taken from the aortic arch (AO) and BCA in mice with fat feeding and saline, mice with fat feeding and PAG treatment. (B) Representative UBM images were taken from CCA in mice with fat feeding and saline, mice with fat feeding and PAG treatment. Scale bar for UBM  =  1 mm. Atherosclerotic plaques are identified by arrows. (C) Representative H&E staining images were taken from BCA in mice with fat feeding and saline, mice with fat feeding and PAG treatment. Scale bar for histological images  =  100 µm.

Moreover, administration with PAG significantly depleted endogenous H_2_S in fat –fed apoE^−/−^ mice (plasma H_2_S concentration in fat+saline and fat+PAG group: 16.43±2.43 µmol/L vs. 5.32±0.87 µmol/L, P<0.05). As shown in Figure 7 and table 3, PAG treatment significantly exacerbated atherosclerosis in aorta, as characterized by a significant increase in the BCA plaque size, CCA IMT and aortic arch IMT assessed by UBM, as well as an obvious rise in BCA plaque area and lipid core evaluated by histology analysis.

**Table 3:** Effect of PAG on plaque development in aorta by UBM and histology assessment.

	UBM			Histology	
	Aortic arch IMT	CCA IMT	Plaque area in BCA	Plaque area	Lipid core
	(mm)	(mm)	(mm^2^)	(mm^2^)	(%)
Fat + saline	0.1039±0.0087	0.0946±0.0060	0.1164±0.0086	0.1073±0.0092	28.65±3.06
Fat + PAG	0.1343±0.0092*	0.1236±0.0074*	0.1470±0.0087*	0.1368±0.0099*	37.48±2.02*

* P<0.05, vs. fat + saline group;

In addition, treatment with NaHS or PAG did not affect body weight or the concentrations of plasma lipids but resulted in a slight reduction in blood pressure (Table S5 and S6).

### Effect of H_2_S on CX3CR1 and CX3CL1 Expression in vivo

**Figure 8:**
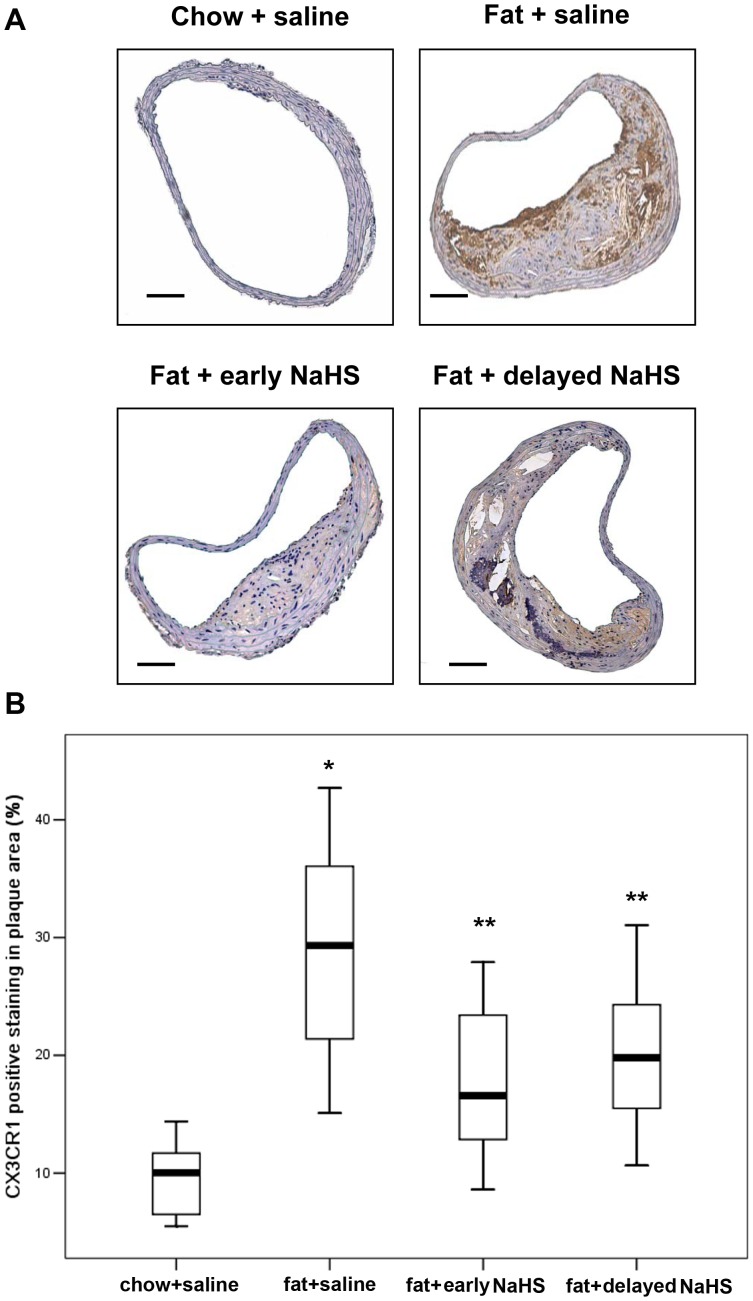
Effect of NaHS on the expression of CX3CR1 in atherosclerotic plaques in fat-fed apoE^−/−^ mice. (A) Representative immunohistochemical images were taken from BCA in mice with chow and saline, mice with fat feeding and saline, mice with fat feeding and early NaHS treatment or mice with fat feeding and delayed NaHS treatment. Scale bar for histological images  =  100 µm. (B) BCA sections from mice with chow and saline, mice with fat feeding and saline, mice with fat feeding and early NaHS treatment or mice with fat feeding and delayed NaHS treatment, were quantified immunohistochemically for CX3CR1 positive staining. Results shown are the mean ± SEM (n = 6–8 animals in each group). *P<0.05, compared with mice with chow and saline; **P<0.05, compared with mice with fat feeding and saline.

**Figure 9:**
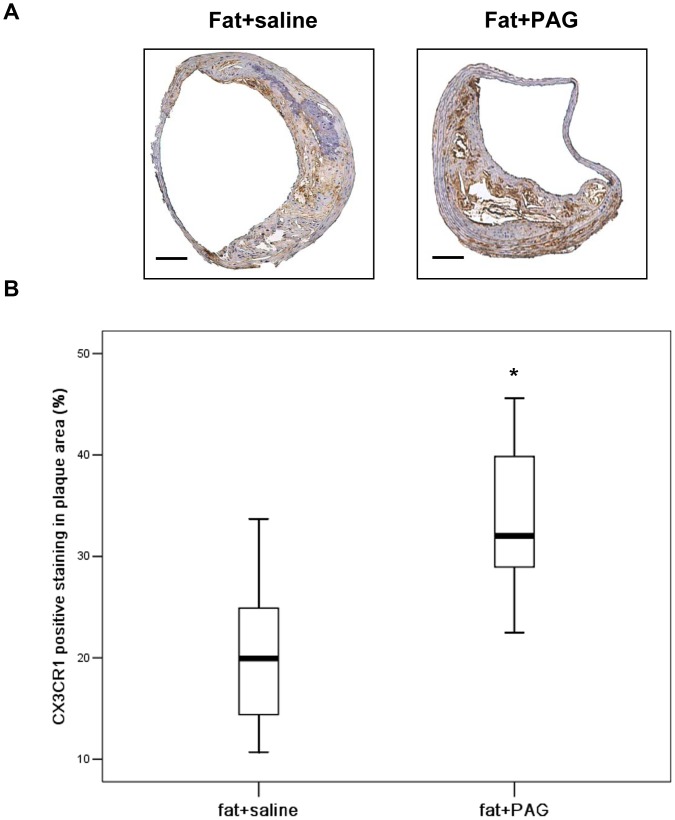
Effect of PAG on the expression of CX3CR1 in atherosclerotic plaques in fat-fed apoE^−/−^ mice. (A) Representative immunohistochemical images were taken from BCA in mice with fat feeding and saline, mice with fat feeding and PAG treatment. Scale bar for histological images  =  100 µm. (B) BCA sections from mice with fat feeding and saline and mice with fat feeding and PAG treatment were quantified immunohistochemically for CX3CR1 positive staining. Results shown are the mean ± SEM (n = 6–8 animals in each group). *P<0.05, compared with mice with fat feeding and saline.

**Figure 10:**
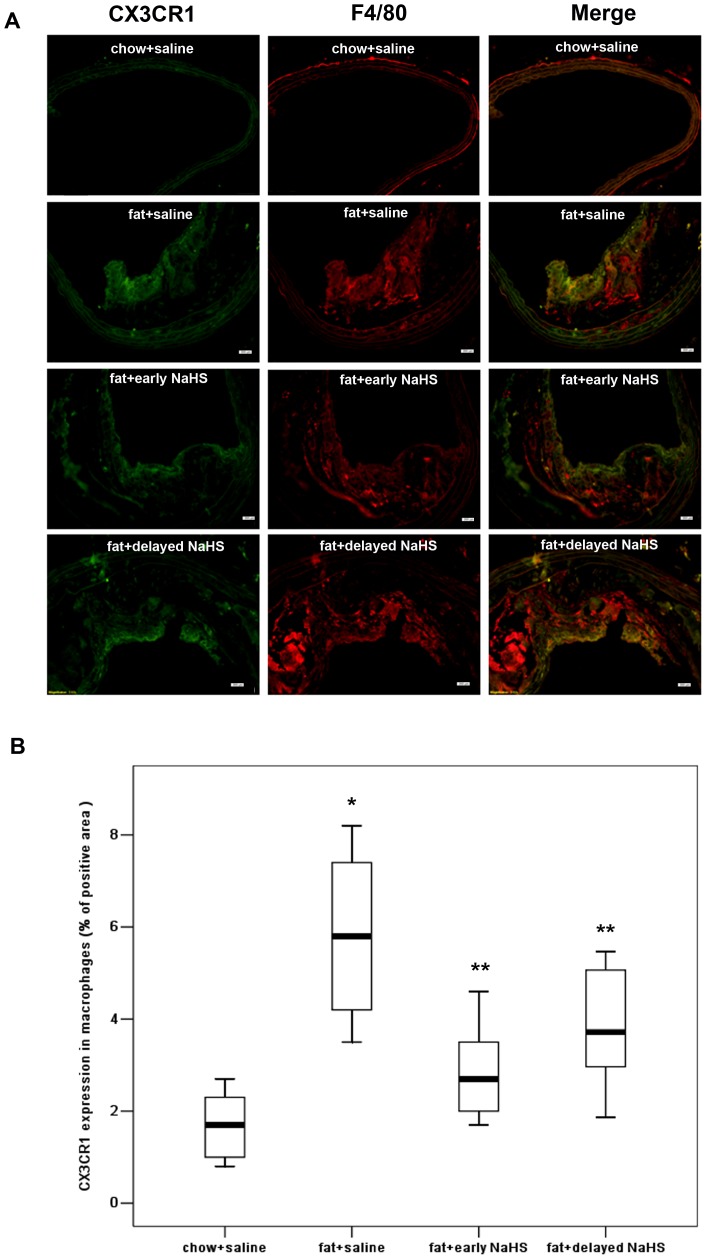
Effect of NaHS on the expression of CX3CR1 in macrophages in atherosclerotic plaques. (A) Representative immunofluorescent stainging images for CX3CR1 and F4/80 colocalization were taken from BCA in mice with chow and saline, mice with fat feeding and saline, mice with fat feeding and early NaHS treatment or mice with fat feeding and delayed NaHS treatment. Scale bar for histological images  =  20 µm. (B) BCA sections from mice with chow and saline, mice with fat feeding and saline, mice with fat feeding and early NaHS treatment or mice with fat feeding and delayed NaHS treatment, were quantified immunohistochemically for CX3CR1 positive staining. Results shown are the mean ± SEM (n = 6–8 animals in each group). *P<0.05, compared with mice with chow and saline; **P<0.05, compared with mice with fat feeding and saline.

**Figure 11:**
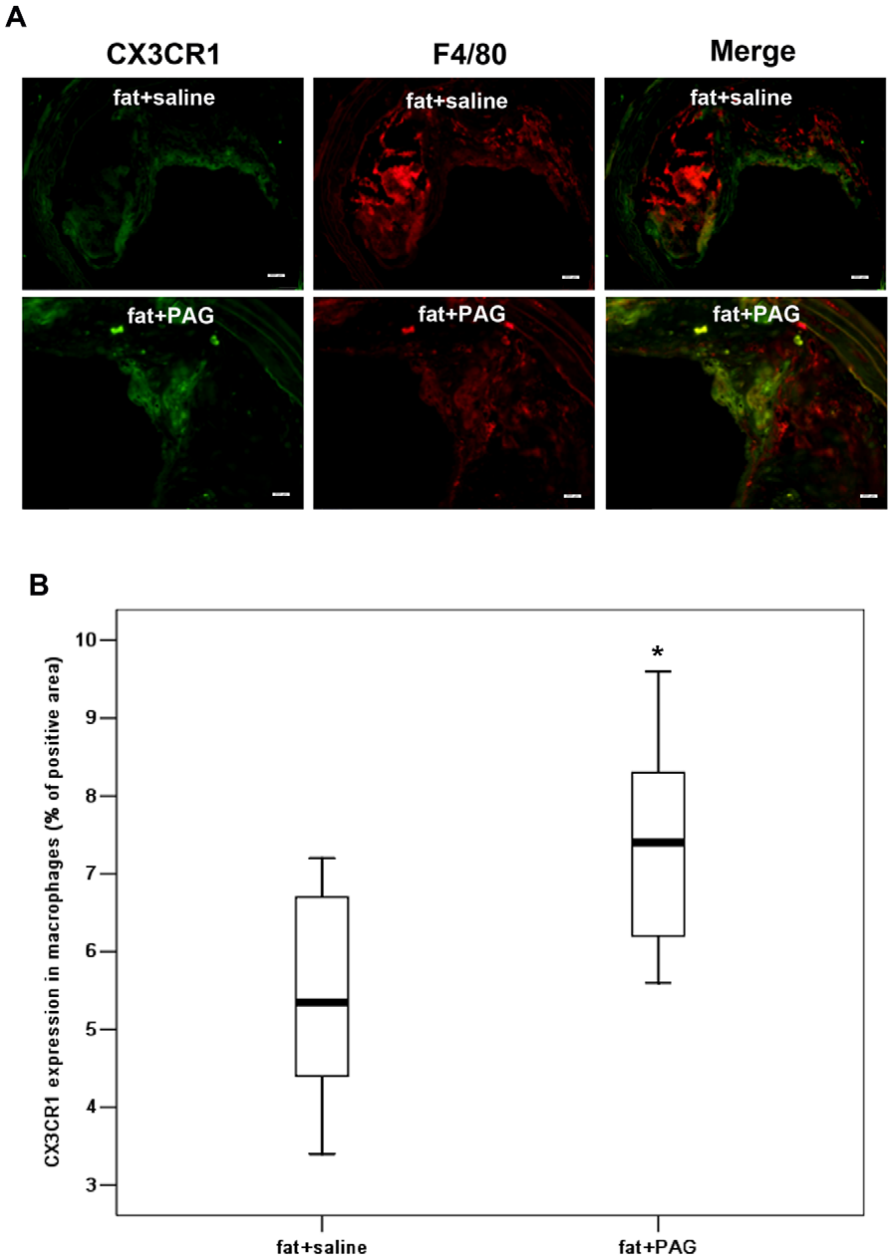
Effect of PAG on the expression of CX3CR1 in macrophage in atherosclerotic plaques. (A) Representative immunofluorescent staining images for CX3CR1 and F4/80 colocalization were taken from BCA in mice with fat feeding and saline, mice with fat feeding and PAG treatment. Scale bar for histological images  =  20 µm. (B) BCA sections from mice with fat feeding and saline and mice with fat feeding and PAG treatment, were quantified immunohistochemically for CX3CR1 positive staining. Results shown are the mean ± SEM (n = 6–8 animals in each group). *P<0.05, compared with mice with fat feeding and saline.

Immunohistochemistry revealed that CX3CR1 was widely expressed in macrophages and foam cells in atherosclerotic plaques (Figure 8A). Both early and delayed treatment with NaHS suppressed the expression of CX3CR1 in atherosclerotic plaques (Figure 8). Early NaHS treatment induced a greater reduction in the CX3CR1 expression in atherosclerotic plaques compared with delayed NaHS treatment (Figure 8). However, PAG treatment upregulated CX3CR1 expression in plaques (Figure 9). Furthermore, as shown in Figure 10 and 11, CX3CR1 staining predominantly colocalized in macrophages within plaques, suggesting that alterations in CX3CR1 expression in plaque lesions are mainly caused by macrophages and that H_2_S may directly act on plaque macrophages to lower CX3CR1 expression in vivo (Figure 8S, negative control for immunofluorescent staining).

Moreover, both early and delayed NaHS inhibited aortic CX3CL1 protein expression in fat-fed apoE^−/−^ mice (Table 4). Early NaHS resulted in a greater reduction in aortic CX3CL1 expression in comparison to delayed NaHS treatment (Table 4). However, PAG significantly elevated aortic CX3CL1 expression (Table 5). Neither NaHS nor PAG had effect on plasma CX3CL1 level (Table 4 and 5).

**Table 4:** Effect of NaHS on CX3CL1 in vivo.

	Plasma CX3CL1 (ng/ml)	Aortic CX3CL1 (ng/ml)
		Aortic protein level (mg/ml)
chow + saline	4.38±1.03	1.36±0.18
Fat + saline	10.32±1.08*	2.82±0.31*
Fat + early NaHS treatment	11.29±1.04	1.60±0.12**#
Fat + delayed NaHS treatment	9.45±0.96	2.15±0.13**

* P<0.05, vs. chow + saline group;

** P<0.05, vs. fat + saline group;

# P<0.05, vs. fat+delayed NaHS treatment group.

**Table 5:** Effect of PAG on CX3CL1 in vivo.

	Plasma CX3CL1(ng/ml)	Aortic CX3CL1(ng/ml)
		Aortic protein level (mg/ml)
Fat + saline	8.73±0.91	2.06±0.17
Fat + PAG	9.69±1.01	1.43±0.13*

* P<0.05, vs. fat + saline group.

In addition, we used flow cytometry to examine the effect of H_2_S on CX3CR1 expression on circulating mouse monocytes. Treatment with NaHS or PAG in fat-fed apoE^−/−^ mice had negligible effect on CX3CR1 expression on CD11b^+^ mononuclear cells (Figure S9A and S9B). As a result, H_2_S did not impact CX3CR1-mediated chemotaxis of mononuclear cells isolated from peripheral blood of hyperlipidemic mice (Figure S9C).

### Effect of H_2_S on CCR2, CCR5, CCL2 and CCL5 Expression in vivo

As shown in Table S7 and Table S8, both NaHS and PAG had minor effect on aortic CCR2, CCR5 mRNA expression and plasma CCL2 and CCL5 concentration in fat-fed apoE^−/−^ mice.

## Discussion

We have shown here that H_2_S synthesis was decreased in a time-dependent manner during the development of atherosclerosis. In fat-fed apoE^−/−^ mice, H_2_S synthesis activity and CSE expression in the aorta gradually decreased and remained extremely low during the late stage of atherosclerosis. As a result, plasma H_2_S concentration began to decline as early as four weeks after high-fat feeding, followed by a significant decrease 8 weeks after fat feeding, and thereafter remained at a low level. The disturbance of the CSE/H_2_S pathway in atherosclerosis was also reported in other studies. Wang et al. first found that the plasma H_2_S level and H_2_S production rate in the aorta were significantly decreased in apoE^−/−^ mice [4]. CSE expression and H_2_S production were reduced during the development of balloon injury-induced neointimal hyperplasia [6]. Taken together, these findings demonstrate that endogenous H_2_S deficiency in the aorta may accompany the pathogenesis of atherosclerosis. These data provide a solid basis for the use of H_2_S donor drugs in the treatment of atherosclerosis. Although the present study is not the first to address the direct correlation between H_2_S and atherosclerosis, our observation on the dynamic alterations of H_2_S metabolism during atherosclerosis may be helpful to identify the right time point for starting preventive or therapeutic interventions for atherosclerosis.

The critical role of H_2_S in the pathogenesis of atherosclerosis is further highlighted by experiments in which insufficiency of endogenous H_2_S formation was manipulated by NaHS, an H_2_S donor. NaHS treatment was given to mice either at the early stage of atherosclerosis or was delayed until the plaques were fully established. Aortic plaque burden was evaluated by both UBM technology and histological analysis. It was found that NaHS significantly reduced plaque size and IMT in the aortic arch and the main branches of the aortic arch. The anti-atherogenic effect of NaHS was more potent when the treatment was commenced at the early stage of atherosclerosis rather than at the advanced stage. Furthermore, depletion of endogenous H_2_S formation by PAG treatment significantly aggravated atherosclerotic lesions in aorta. These interesting observations reinforce the view that H_2_S is involved in the initiation and progression of atherosclerosis. They also clearly suggest that prevention of atherosclerosis is correlated with a supplement of exogenous H_2_S and that H_2_S may exert a more prominent anti-atherosclerotic effect when it is applied during the initial stage of lesion formation.

H_2_S has been shown to be endogenously generated in monocytes and macrophages and in turn plays an important role in regulating the biological functions of these cells [25], [26]. H_2_S inhibited LPS-induced NO production and alpha-tumor necrosis factor (TNF-α) secretion in microglia, a specialized form of macrophages residing in central nervous systems [27]. Zhi et al. reported that H_2_S stimulated the activation of human monocytes with the generation of cytokines [25]. However, to date, the effect of H_2_S on macrophage recruitment and hemostasis in atherosclerotic lesions has not been determined. Recently, chemokines (CCL2, CCL5 and CX3CL1) and chemokine receptors (CCR2, CCR5 and CX3CR1) have been strongly implicated to play critical roles in mediating inflammation, macrophage recruitment and atherosclerosis progression by multiple lines of evidence, including expression, functional and epidemiologic data [8]–[20]. Therefore, we performed a series of in vitro experiments to investigate the role of H_2_S in regulating CCL2-CCR2, CCL5-CCR5 and CX3CL1-CX3CL1 expression in RAW264.7 cells and mouse peritoeal macrophages. Activation of macrophages was achieved by classic stimulators, IFN-γ and LPS. Cell activation resulted in enhanced CX3CL1 and CX3CR1 expression and chemotaxis towards CX3CL1. Supplement of exogenous H_2_S dose-dependently reduced the LPS or IFN-γ induced-upregulation of CX3CL1 and CX3CR1 in macrophages. Similarly, overproduction of endogenous H_2_S via CSE overexpression also inhibited CX3CL and CX3CR1 expression in stimulated macrophages. As a result, either exogenous applied H_2_S or overexpression of CSE reduced macrophage migration in response to CX3CL1 after stimulation with IFN-γ or LPS. Collectively, our findings demonstrate that H_2_S plays a crucial role in regulating CX3CL1 and CX3CR1 expression and CX3CR1-mediated chemotaxis in stimulated macrophages.

To further demonstrate the involvement of H_2_S in regulating CX3CL1 and CX3CR1 expression during atherosclerosis and to establish its pathophysiological relevance in vivo, we examined the effect of H_2_S on CX3CL1 and CX3CR1 expression in fat-fed apoE^−/−^ mice. Administration of NaHS at either the early or the advanced stage of atherosclerosis suppressed the aortic expression of CX3CL1 and CX3CR1, together with reduced plaque size and IMT thickness in the main branches of the aortic arch. In contrast, inhibition of endogenous H_2_S formation upregulated aortic expression of CX3CL1 and CX3CR1, thus exacerbating atherosclerosis in aorta. Furthermore, immunofluorescent staining revealed that CX3CR1 colocalized with macrophages, suggesting that the reduction in plaque CX3CR1 expression is predominantly by macrophages and that other cell types are likely to play insignificant roles. These findings indicated that H_2_S may directly act on plaque macrophages and inhibit CX3CR1 expression in vivo. These results are consistent with the findings of in vitro studies with mouse macrophage cell line or isolated mouse peritoneal macrophages. However, flow cytometry analysis found that injection of NaHS or PAG did not alter CX3CR1 expression on circulating monocytes isolated from fat-fed aopE^−/−^ mice. This lack of change may be due to the short half-life (approximately 6 hours) of circulating monocytes, such that their exposure time to H_2_S is inadequate to inhibit CX3CR1 expression. This result also supports the concept that H_2_S induced reduction in CX3CR1 expression occurs in macrophages within plaques but not in the circulating monocytes. A decline in CX3CR1 expression in macrophages at the site of lesions may be beneficial, eg suppressing the recruitment and retention of macrophages in blood vessel walls and thereby retarding the development and progression of atherosclerosis.

The present study found that H_2_S inhibited CX3CL1 expression in stimulated macrophages and in aorta tissues. However, it had no effect on plasma CX3CL1 level in vivo. It may be attributed to the fact that CX3CL1 is predominantly a membrane-bond chemokine. Furthermore, H_2_S did not affect CCL2-CCR2 and CCL5-CCR5 expression both in vitro and in vivo. These observations support the notion that CCL2-CCR2, CCL5-CCR5 and CX3CL1-CX3CR1 axis impact the pathogenesis of atherosclerosis independently.

Recent studies have shown that PPARγ has the inherent capacity to regulate expression of macrophage chemokine receptors CX3CR1 in the lipid-mediated inflammation process of atherosclerosis lesions [21], [22]. Wan et al. identified a direct link between PPARγ and the CX3CL1/CX3CR1 signaling [28]. Activation of PPARγ not only downregulated CX3CR1 gene transcription but also prevented plasma membrane translocation of CX3CR1 protein [28]. Therefore, we investigated the underlying mechanism by which H_2_S regulates CX3CR1 expression and CX3CR1-mediated chemotaxis in macrophages with respect to the PPARγ pathway. Here, we showed that H_2_S-induced inhibition of CX3CR1 expression in stimulated macrophages is dependent on PPAR-γ activation. Support for this concept comes from the observation that NaHS dose-dependently increased the DNA binding activity of PPAR-γ in RAW264.7 cells and mouse peritoneal macrophages stimulated with IFN-γ or LPS. CSE overexpression also enhanced the activation of PPAR-γ in IFN-γ or LPS stimulated RAW264.7 cells. Moreover, our notion regarding the involvement of PPAR-γ was further strengthened by the finding that blockage of PPAR-γ activation by GW9662 markedly reduced the inhibition of CX3CR1 expression induced by NaHS or CSE overexpression in stimulated macrophages. Consequently, GW9662 eliminated exogenous or endogenous H_2_S-induced inhibition of CX3CR1-mediated chemotaxis in activated macrophages. Together, these results indicate that PPAR-γ is possibly involved in the signaling transduction of H_2_S-induced inhibition of CX3CR1 expression in macrophages.

H_2_S is a highly reactive molecule that readily reacts with free reactive oxygen species (ROS) and reactive nitrogen species (RNS) [29]–[31]. All these compounds react rapidly to form peroxynitrite, which then attacks tyrosine residues in proteins to form nitrotyrosine. Nitration of PPAR-γ following inflammatory stimulation in RAW 264.7 cells inhibited its ligand-dependent translocation into the nucleus [32]. These findings raise the possibility that H_2_S may suppress the nitration of PPAR-γ by directly scavenging ROS and/or RNS, thus provoking the activation of PPAR-γ and downregulating the expression of CX3CR1 in macrophages. However, the precise mechanism by which H_2_S regulates the activation of PPAR-γ in stimulated macrophages is still far from clear. Further investigations are required to address this and to resolve the detailed structural basis for PPAR-γ induction of CX3CR1 expression.

CX3CL1 expression has been shown to be modulated by NF-κB activation [24]. Therefore, we investigated the involvement of NF-κB pathway in regulating CX3CL1 expression by H_2_S. The present study found that H_2_S inhibited IκB degradation, the key step to NF-κB nuclear translocation and activation, and decreased the DNA binding activity of NF-κB p65 in macrophage nuclear extracts. This suggests that H_2_S may downregulate CX3CL1 expression in macrophages by suppressing the activation of NF-κB pathway.

Finally, it is to be noted that our data are still insufficient to establish a clear causal link between the inhibitory effect of H_2_S on athoerslcerosis and macrophage CX3CR1-CX3CL1 expression. Further work is needed most likely in CX3CR1/CX3CL1 gene modified mice to probe the exact linkage involved. In addition to its effect on the macrophage CX3CR1-CX3CL1 expression, H_2_S has been shown to impede the pathogenesis of atherosclerosis in various ways [33]. For instance, H_2_S plays a role in regulating vascular smooth muscle cell (VSMC) proliferation and apoptosis. It also inhibits atherogenic modification of low-density lipoprotein (LDL), interferes with vascular calcification and is involved in platelet function.

In summary, the present study suggests that deficiency of endogenous H_2_S formation in the aorta accompanies the development and progression of atherosclerosis. H_2_S may have an anti-atherogenic benefit and this effect seems to be more potent when it is applied during the early stage of atherosclerosis. H_2_S may prevent the progression of atherosclerosis, along with downregulating macrophage CX3CR1 and CX3CL1 expression by a PPAR-γ and NF-κB dependent mechanism. Our study therefore provides evidence that H_2_S donors may be a possible new therapeutic strategy to counteract the development of atherosclerosis or other immune diseases.

## Materials and Methods

### Cell Culture

RAW 267.4 murine macrophage cells were obtained from ATCC. Cells were grown in Dubbelco’s Modified Eagle’s medium (DMEM) containing 10% fetal bovine serum (FBS) (Invitrogen, Carlsbad, CA, USA), 100 units/ml penicillin, and 100 mg/ml streptomycin and plated on 6-well plates or 100 mm tissue culture dishes 24 h before experiments. Near-confluent cultures were switched to medium containing 0.5% FBS for 24 h before NaHS treatment or stimulation with LPS or IFN-γ. No cells older than passage 10 were used, because RAW cells showed highly variable responses at higher passage numbers.

### Macrophage Isolation

Peritoneal macrophages were isolated from 10-week, male C57BL/6J mice. A 3% thioglycolate solution (Sigma-Aldrich, St Louis, MO, USA) was injected intraperitoneally and, 4 days later, peritoneal macrophages were harvested through phosphate-buffered saline lavage. Ten milliliters of ice-cold phosphate-buffered saline was injected into the mouse peritoneal cavity and gently removed. Once collected, cells were seeded in RPMI medium 1640 supplemented with 10% fetal bovine serum (Invitrogen) and 1% penicillin/streptomycin for 4 h and then rinsed and maintained in RPMI medium 1640 supplemented with 0.5% FBS for 24 h before NaHS treatment or stimulation with LPS or IFN-γ.

### Cell Treatment

Cells were rinsed twice with serum-free culture medium before treatment and all treatments were carried out in serum-free culture medium containing penicillin and streptomycin. Cells were pre-incubated with saline or NaHS (50, 100 and 200 µM) for 6 hours and then stimulated with IFN-γ (500 U/ml) or LPS (10 µg/ml) for 12 hours in the continuous presence of NaHS. For experiments using inhibitors, cells were pre-incubated with GW9662 (10 µM) for 1 hour before NaHS addition.

### Animal Experiments

The study was carried out in strict accordance with the recommendations in the Guide for the Care and Use of Laboratory Animals of the Shanghai JiaoTong University School of Medicine. The protocol was approved by the Committee on the Ethics of Animal Experiments of the Shanghai JiaoTong University School of Medicine (Permit Number: [2011]-23). C57BL/6J male apoE^−/−^ mice were purchased from the Animal Center of the Beijing University, Beijing, China. At the age of 8 weeks, feeding of animals on a high-fat diet began that contained 21% fat from lard and was supplemented with 0.15% (w/w) cholesterol. At indicated time points (0, 4, 8, 12, 24 weeks after fat feeding), mice were sacrificed by an intraperitoneal (i.p.) injection of a lethal dose of pentobarbitone. To investigate the role of H_2_S in atherosclerosis, different drug administration schedules were used. In the early-NaHS treatment study, thirty mice began to receive NaHS (1 mg/kg, i.p., daily) 4 weeks after fat feeding and were sacrificed 24 weeks after fat feeding. In the delayed-NaHS treatment study, thirty mice began to receive NaHS (1 mg/kg, i.p., daily) 12 weeks after fat feeding and were sacrificed 24 weeks after fat feeding. In DL-propargylglycine (PAG) treatment study, eighteen mice began to receive PAG (10 mg/kg, i.p., daily) 4 weeks after fat feeding and were sacrificed 12 weeks after fat feeding. Samples of aortas and plasma were harvested and stored at –80°C for subsequent measurement.

Systolic blood pressure (SBP) was measured at the beginning and the end of the studies by using a non-invasive computerized tail cuff system (Blood Pressure Analysis System BP- 98AW monitor, Softron Co. Ltd., Tokyo, Japan). A series of readings was taken and an average of 3–5 BP readings was used. Blood samples were drawn from the left ventricle of mice at the end of the studies and plasma lipid concentrations were determined in heparinized plasma.

### Cloning of CSE cDNA and Transient Transfection

PCR was used to amplify the open reading frame of CSE (GenBank^TM^ accession number: NM_145953) from C57BL/6J mouse liver using the primers 5′-CAT GCA GAA GGA CGC CTC TTT GA-3′ and 5′-TTA AGG GTG CGC TGC CTT CAA AGC -3′. The amplified open reading frame of CSE was subcloned into the TA cloning vector (PCR^®^2.1-TOPO, Invitrogen). A positive clone containing CSE open reading frame insert was sequenced to confirm the accuracy of the inserted CSE sequence. The constructs containing CSE cDNA were cleaved and subcloned into the mammalian expression vector pcDNA3.1 (Invitrogen). The CSE expression vectors or the control vectors (pcDNA3.1 empty vectors) were transiently transfected into RAW264.7 cells using FuGENE HD Transfection Reagent (6 µl:2 µg reagent to plasmid DNA ratio, Roche, Switzerland), according to manufacturer’s instruction.

### RT-PCR

Total RNA from cells or tissues was extracted with Trizol ® reagent (Invitrogen) according to the manufacturer’s protocol. The concentration of isolated nucleic acids was determined spectrophotometrically by measuring the absorbance at 260 nm and the integrity was verified by ethidium bromide staining of 18 S and 28 S rRNA bands on a denaturing agarose gel. All samples were thereafter stored at −80°C until required. 1 µg of RNA was reversely transcribed using an iScript™ cDNA Synthesis Kit (Bio-Rad Laboratories, Hercules, CA, USA) at 25°C for 5 minutes, 42°C for 30 minutes, followed by 85°C for 5 minutes. The cDNA was used as a template for PCR amplification by iQ™ Supermix (Bio-Rad Laboratories). The primer sequences, optimal annealing temperature, optimal cycles and product sizes are as shown in Table 6. PCR amplification was carried out in a MyCycler™ system (Bio-Rad Laboratories). The reaction mixture was first subjected to 95°C for 3 minutes, followed by an optimal cycle of amplifications, consisting of 95°C for 50 s, optimal annealing temperature for 45 s and 72°C for 1 minute. PCR products were analyzed on 1.5 % w/v agarose gels containing 0.5 µg/ml ethidium bromide.

**Table 6:** PCR primer sequences, optimal conditions and product sizes.

Gene	Primer sequence	Optimal conditions	Size
CSE	Forward:5′-GAC CTC AAT AGT CGG CTT CGT TTC-3′Reverse:5′-CAG TTC TGC GTA TGC TCC GTA ATG-3′	34cyclesAnnealing: 61°C	618 bp
CX3CR1	Forward:5′-CCT GCC TCT GAG AAA TGG AG -3′Reverse: 5′- ATC TCT CCA GCC CCT GAA AT -3′	36 cyclesAnnealing: 58°C	332 bp
CX3CL1	Forward:5′-ATG ACC TCA CGA ATC CCA GTG -3Reverse: 5′-CCG CCT CAA AAC TTC CAA TGC -3′	36 cyclesAnnealing: 60°C	453
CCR2	Forward: 5′-AGAGAGCTGCAGCAAAAAGG-3′Reverse: 5′-GGAAAGAGGCAGTTGCAAAG-3	35 cyclesAnnealing: 56°C	185 bp
CCR5	Forward: 5′-CGAAAACACATGGTCAAACG-3′Reverse: 5′- CATGGCACCTGCCTCAAGTCTC-3′	36 cyclesAnnealing: 52°C	364 bp
GAPDH	Forward: 5′-GCA CAG TCA AGG CCG AGA AT -3′Reverse: 5′-GCC TTC TCC ATG GTG GTG AA -3′	22cyclesAnnealing: 54°C	151 bp

### Quantification of Chemokine Concentrations by ELISA

CCL2, CCL5 and CX3CL1 protein levels were assayed in aorta and cell homogenates as well as mouse plasma and culture media using commercially available ELISA kits (Quantikine, R&D systems).

### Western Immunoblot

Cultured cells (3×10^6^) were harvested and lysed at 4°C in radioimmunoprecipitation assay lysis buffer. Aorta was homogenized at 4°C in radioimmunoprecipitation assay lysis buffer. The cell lysate or tissue homogenates were clarified by centrifugation at 14,000×g for 10 min at 4°C. Protein concentration in the soluble fraction was determined by the Bradford method. Protein samples (50–100 µg) were separated by a 12% SDS-polyacrylamide gel and then transferred onto polyvinylidene difluoride membranes. Membranes were then washed, blocked, and probed overnight at 4°C with rabbit polyclonal anti-CX3CR1 (1∶1000, Abcam, USA), IκBα (1∶1000, Cell Signaling Technology, USA), β-actin antibodies (1∶2000, Santa Cruz Biotechnology, USA) and mouse monoclonal anti-CSE antibody (1∶1000, Abnova, Taiwan) respectively, followed by secondary antibody for 2 h with a 1∶2000 dilution of HRP-conjugated, goat anti-rabbit IgG or goat anti-mouse IgG (Santa Cruz Biotechnology). Membranes were washed and then incubated in SuperSignal™ West Pico chemiluminescent substrate (Pierce, USA) before exposure to X-ray films (Pierce). The intensity of bands was quantified using LabWorks™ Image Analysis software (UVP).

### Flow Cytometry

Male apoE^−/−^ were given saline, NaHS (1 mg/kg, i.p., daily) or PAG (10 mg/kg, i.p., daily) at the same time as the commencement of fat feeding and were sacrificed 8 weeks after fat feeding. Mononuclear cell (MNC) fraction was isolated from 1 ml of whole mouse blood using centrifugation (400 g, 30 min) with Ficoll-Paque Plus (Amersham Biosciences, Sweden) as described previously [34]. After washing with PBS, monocytes (1×10^6^/tube) were labelled with rabbit anti-mouse CX3CR1 antibody for 1 h at room temperature, followed by incubation for a further 45 min with secondary PE-labelled antibodies (BD Biosciences) at room temperature. A third 30 min incubation was then carried out with a FITC conjugated CD11b antibody (BD Pharmingen). Appropriate anti-rabbit IgG control (Abcam) and Rat IgG control (BD Pharmingen) were also tested for CX3CR1 and CD11b antibodies, respectively. CX3CR1^+^CD11b^+^ cells were identified by flow cytometry (BD FACSCalibur Flow Cytometer) using FlowJo 5.7.2 software. For each sample, 100 000 cells were acquired.

### Chemotaxis Assay

Chemotaxis was evaluated with QCM™ Chemotaxis 5 µm 96-well Cell Migration Assay (Chemicon, US). Serum free medium or chemoattractant solution (150 µL) was added to the lower chamber and 100 µL of cell suspension (2×10^6^/ml) was added to the upper compartment. The cell migration plate assembly was incubated at 37°C with 5% CO_2_ for 2 h. Migratory cells on the bottom of insert membrane were dissociated from the membrane when incubated with Cell Detachment Buffer. The cells that migrated into the medium in the lower chamber or detached from the membrane by Cell Detachment Buffer were subsequently lysed and detected by CyQuant GR dye. This green fluorescent dye exhibits strong fluorescence enhancement when bound to cellular nucleic acids. The intensity of fluorescence, which correlated with the number of migrated cells, was measured with a fluorescence plate reader using a 485/520 nm filter set. Results were expressed as fluorescence intensity, RFU (relative fluorescence unit), by subtracting background fluorescence in cells migrating to the medium without chemoattractant, from specific fluorescence in cells migrating to CX3CL1.

### Transcription Activity of PPAR-γ and NF-κB

Nuclear extracts from RAW264.7 or peritoneal cells were prepared using a Nuclear Extraction Kit, as described by the manufacturer (Active Motif, Tokyo, Japan). Protein concentrations in nuclear extracts were determined using the Bradford assay (Bio-Rad Laboratories). PPAR-γ or NF-κB p65 DNA binding activity in nuclear extracts was assayed in triplicate with TransAM PPAR-γ Transcription Factor Assay Kits (Active Motif) according to manufacturer’s instructions. The OD_450_ was read on a 96-well microplate reader (Tecan Systems Inc.).

### Measurement of Plasma H_2_S

Aliquots (120 µl) of plasma were mixed with distilled water (100 µl), trichloroacetic acid (10% w/v, 120 µl), zinc acetate (1% w/v, 60 µl), N, N-dimethyl-p-phenylenediamine sulfate (20 µM; 40 µl) in 7.2 M HCl and FeCl_3_ (30 µM; 40 µl) in 1.2 M HCl. The absorbance of the resulting solution (670 nm) was measured 10 min thereafter by spectrophotometry (Tecan Systems Inc.). H_2_S was calculated against a calibration curve of sodium hydrosulfide (NaHS; 3.125–100 µM). Results were shown as plasma H_2_S concentration in µM.

### Measurement of Plasma H_2_S by Sulfur-sensitive Electrode

Plasma H_2_S was measured by a sulfur-sensitive electrode (PXS-270, Shanghai, China) [4]. Briefly, 0.2 mL of plasma was mixed with 0.2 mL of antioxidant buffer. After being rinsed with distilled water and dried, the electrode was immersed into the plasma. Electrode potential was recorded when the reading stabilized. H_2_S was calculated against a calibration curve of sodium hydrosulfide (NaHS; 3.125–100 µM). Results were shown as plasma H_2_S concentration in µM.

### H_2_S Synthesizing Activity Assay

H_2_S synthesiszing activity in aorta homogenates was measured essentially as described elsewhere [35]. Briefly, the assay mixture contained 100 mM potassium phosphate buffer (pH 7.4), L-cysteine (20 µl, 20 mM), pyridoxal 5′-phosphate (20 µl, 2 mM), saline (30 µl) and 4.5% w/v tissue homogenate (430 µl). The reaction was performed in tightly sealed micro centrifuge tubes and initiated by transferring the tubes from ice to a water bath at 37°C. After incubation for 30 min, 1% w/v zinc acetate (250 µl) was added to trap evolved H_2_S, followed by 10% v/v trichloroacetic acid (250 µl) to denature the protein and stop the reaction. Subsequently, N, N-dimethyl-p-phenylenediamine sulfate (20 µM; 133 µl) in 7.2 M HCl was added, immediately followed by FeCl_3_ (30 µM; 133 µl) in 1.2 M HCl. The absorbance of the resulting solution at 670 nm was measured by spectrophotometry (Tecan Systems Inc). The H_2_S concentration was calculated against a calibration curve of NaHS. Results were then corrected for the DNA content of the tissue sample and expressed as nmoles H_2_S formed/mg DNA.

### Ultrasound Biomicroscopy

Ultrasound biomicroscopy was performed as previously described [36], [37]. Briefly, mice were lightly anesthetized with isoflurane gas. A UBM system (Vevo 770, Visualsonics, Toronto, Canada) equipped with a 40 MHz mechanical transducer was used for all the examinations. A right parasternal long-axis view was employed to visualize the ascending aorta, aortic arch and the neck vessels in one plane. A parasternal short-axis view was taken to visualize the same arterial site in a cross-sectional view immediately proximal to the branch of the brachiocephalic artery. By adjusting the distance between the transducer and the target vascular site of interest, the intima-media complex thickness (IMT) and atherosclerotic lesions can be readily visualized. Thereafter, during diastole, an optimal freeze-frame image was taken manually to visualize the plaque area by using the leading-to-leading edge approach. IMT was measured from the leading echo edge (intima) closest to the blood stream to the next leading echo edge representing the adventitial layer. Plaque area was delineated in the cross-sectional view also by using the leading-to-leading edge approach, which also includes the underlying smooth muscle cell layers in the plaque area measurement. In vivo length of the internal elastic lamina was approximated by delineation of the lumen circumference. A 10 s CINE loop was stored digitally for off-line image analysis using an image analysis system (Visualsonics, Toronto, Canada). All measurements were repeated twice at the same site. All images were analyzed by an operator blinded to the identities of the animals.

### Histological Examination

Mice were anesthetized, followed by left ventricle perfusion with PBS and 10% neutralized formalin at a constant pressure of 100 mmHg. Brachiocephalic arteries were removed with a piece of the aortic arch and the stump of the right subclavian artery still attached to aid orientation during histological processing. Morphometric studies were performed in brachiocephalic artery. Brachiocephalic arteries were embedded in paraffin. Five sections (5 µm) taken 30 µm apart from the brachiocephalic artery of every mouse (starting from the proximal end) were stained with hematoxylin and eosin and then used for histological analysis (n = 6 mice in each group). Estimation of plaque lipid content used a valid method as previously described [38]. The maximum plaque thickness was averaged from three lesion sites around the thickest part of a plaque, approximately 100 µm apart. Every morphological parameter was quantified using computer-assisted morphometry (Leica Q win, Heidelberg, Germany) and performed by an investigator blinded for the treatment.

### Immunohistochemistry of CX3CR1

Brachiocephalic arteries were deparaffinized in xylene and rehydrated in aqueous solutions with decreasing alcohol content, followed by a wash in PBST (1×PBS with 0.5% Tween 20, pH 7.4). Antigen retrieval was achieved by heating the slides in 10 mM sodium citrate (pH 6.0) at 95°C for 20 min. Slides were cooled for 20 min and washed in H_2_O and PBST. Endogenous peroxidase activity was inactivated by treatment in 3% H_2_O_2_ for 15 min, followed by a wash in PBST. Nonspecific staining was blocked by incubation with Ultra-V block (Labvision) for 5 min. Slides were incubated with rabbit anti-mouse CX3CR1 antibody (Abcam) in 1∶100 dilution for 2 h at 37°C, followed by secondary antibody in a 1∶200 dilution of HRP-conjugated, goat anti-rabbit IgG (Santa Cruz Biotechnology) for 2 h at room temperature. Immunohistochemical staining was developed by exposure to 3, 3′-diaminobenzidine and counterstaining was performed with hematoxylin. The staining was examined by light microscopy using a light microscope (objective lens magnification of ×40; eyepiece magnification of ×10). For fluorescent immunohistochemical staining, sections were incubated with rabbit anti-mouse CX3CR1 antibody (Abcam, 1∶100) and rat anti mouse F4/80 antibody (Santa Cruz, 1∶100) overnight at 4°C, followed by incubation for 2 hour with fluorescently (Alexa Fluor 488 or Alexa Fluor 594) labeled secondary antibodies (Invitrogen, 1∶100). Sections were then mounted and visualized using a fluorescent microscope (Olympus).

### Statistics

The data were expressed as mean ± SEM. The significance of differences among groups was evaluated by analysis of variance (ANOVA) with the post-hoc Tukey’s test when comparing three or more groups. The significance of differences between two groups was evaluated by the Student’s t-test. A P < 0.05 was regarded as statistically significant.

## Supporting Information

Figure S1 Effect of NaHS on CX3CR1 expression in mouse peritoneal macrophages stimulated with IFN-γ or LPS. RT-PCR analysis for CX3CR1 mRNA (A) and western blot analysis (B) for CX3CR1 protein expression were carried out as described in Materials and Methods. The data are means ± SEM of at least three independent experiments. *P<0.05, compared with unstimulated cells (control). ‡P<0.05, compared with stimulated cell pretreated with saline.
(TIF)

Figure S2 Effect of NaHS (100 µM) on chemotactic response to increasing doses of CX3CL1 in RAW264.7 cells stimulated with IFN-γ- or LPS. Chemotaxis of RAW264.7 cells towards CX3CL1 was assayed as described in Materials and Methods. The data are means ± SEM of at least three independent experiments. *P<0.05, compared with saline-pretreated, unstimulated cells (CX3CL1 10 ng/ml). **P<0.05, compared with saline-pretreated, stimulated cells (CX3CL1 10 ng/ml). †P<0.05, compared with saline-pretreated, unstimulated cells (CX3CL1 at the same concentration). ‡P<0.05, saline-pretreated, stimulated cells (CX3CL1 at the same concentration).
(TIF)

Figure S3 Effect of pretreatment with GW9662 on NaHS-induced downregulation of CX3CR1 expression and NaHS-induced inhibition of CX3CR1-mediated chemotaxis in IFN-γ or LPS-stimulated mouse peritoneal macrophages. Cells were incubated with GW9662 (10 µM) or vehicle for 1 hour, further incubated with NaHS (100 µM) or saline for 6 hours and then stimulated with IFN-γ (500 U/ml) or LPS (10 µg/ml) for 12 hours in the continuous presence of NaHS or saline. Western blot analysis for CX3CR1 expression (A) and chemotaxis towards CX3CL1 (50 ng/ml) (B) were assayed as described in Materials and Methods. The data are means ± SEM of at least three independent experiments. *P<0.05, compared with unstimulated cells (control). †P<0.05, compared with stimulated cells treated with saline and vehicle. ‡P<0.05, compared with stimulated cell treated with NaHS and vehicle.
(TIF)

Figure S4 Transfection of CSE cDNA construct in RAW264.7 cell. Cells were transfected with CSE cDNA construct or an identical empty vector lacking a cDNA insert as a control. The expression of CSE was verified by western blot analysis (A) and the H_2_S synthesizing activity (B) was assayed as described in Materials and Methods. The data are means ± SEM of four independent experiments. *P<0.05, compared with control cells. †P<0.05, compared with cells transfected with empty vector.
(TIF)

Figure S5 Effect of NaHS or CSE overexpression on CX3CL1 mRNA expression and IκBα content in macrophages stimulated with IFN-γ or LPS. (A) Mouse peritoneal macrophages were pre-incubated with saline or NaHS (100 µM) for 6 hours and then stimulated with IFN-γ or LPS for 12 hours in the continuous presence of NaHS or saline. (B) RAW264.7 cells were transfected with CSE cDNA construct or empty vector and then were stimulated with IFN-γ or LPS for 12 hours. RT-PCR analysis for CX3CL1 mRNA and western blot analysis for IκBα content were carried out as described in Materials and Methods.
(TIF)

Figure S6 Effect of NaHS on CCR2 and CCR5 mRNA expression in macrophages stimulated with IFN-γ or LPS. RAW264.7 cells (A, B) or mouse peritoneal macrophages (C, D) were pre-incubated with saline or NaHS (50 µM, 100 µM, 200 µM) for 6 hours and then stimulated with IFN-γ or LPS for 12 hours in the continuous presence of NaHS or saline. RT-PCR analysis for CCR2 and CCR5 mRNA was carried out as described in Materials and Methods.
(TIF)

Figure S7 Alterations in plasma H_2_S level during the development of atherosclerosis in fat-fed apoE^−/−^ mice. Plasma H_2_S levels were assayed at indicated time points (0, 4, 8, 12, 24 weeks after fat feeding) by sulfur-sensitive electrode method. Results shown are the mean ± SEM (n = 6 animals in each group). *P<0.05, compared with the basal level at 0 weeks. #P<0.05, compared with mice sacrificed 4 weeks after fat feeding. & P<0.05, compared with mice sacrificed 8 weeks after fat feeding.
(TIF)

Figure S8 Negative control immunofluorescent staining in BCA with mouse IgG1 isotype control and Alexa Fluor 488 or Alexa Fluor 594 labeled secondary antibodies. Scale bar for histological images  =  20 µm.
(TIF)

Figure S9 Effect of H_2_S on CX3CR1 expression and CX3CL1-mediated chemotaxis of circulating monocytes isolated from fat-fed apoE^−/−^ mice. ApoE^−/−^ mice (n = 10 in each group) were fed a high-fat diet and at the same time received saline, NaHS (1 mg/kg, daily, i.p.), or PAG (10 mg/kg, daily, i.p.) and sacrificed 8 weeks after fat feeding. Whole blood was collected and the mononuclear cell fraction was isolated, and then incubated with antibodies to detect CD11b+CX3CR1+ cell populations using flow cytometry (A). CX3CR1 mean fluorescence intensity (B) and CX3CL1-induced chemotaxis (C) were determined among treatment groups. Results are expressed as mean±SEM. *P<0.05, compared with chow+saline group.
(TIF)

Table S1 Effect of NaHS on CX3CL1 level in RAW264.7culture media stimulated with IFN-γ or LPS.
(DOC)

Table S2 Effect of NaHS on CX3CL1 in stimulated mouse peritoneal macrophages.
(DOC)

Table S3 Effect of CSE overexpression on CX3CL1 in stimulated RAW264.7 cells.
(DOC)

Table S4 Effect of H_2_S on CCL2 and CCL5 in stimulated RAW264.7.
(DOC)

Table S5 Effect of treatment with NaHS on blood pressure and plasma lipids.
(DOC)

Table S6 Effect of treatment with PAG on blood pressure and plasma lipids.
(DOC)

Table S7 Effect of H_2_S on CCL2, CCL5, CCR2 and CCR5 in vivo.
(DOC)

Table S8 Effect of PAG on CCL2, CCL5, CCR2 and CCR5 in vivo.
(DOC)
