# Supplementary figures and images for: Hydrogen Sulfide Inhibits the Development of Atherosclerosis with Suppressing CX3CR1 and CX3CL1 Expression
Source: PLoS One. 2012 Jul 18;7(7):e41147. doi: 10.1371/journal.pone.0041147 (PMC3399807; doi:10.1371/journal.pone.0041147)

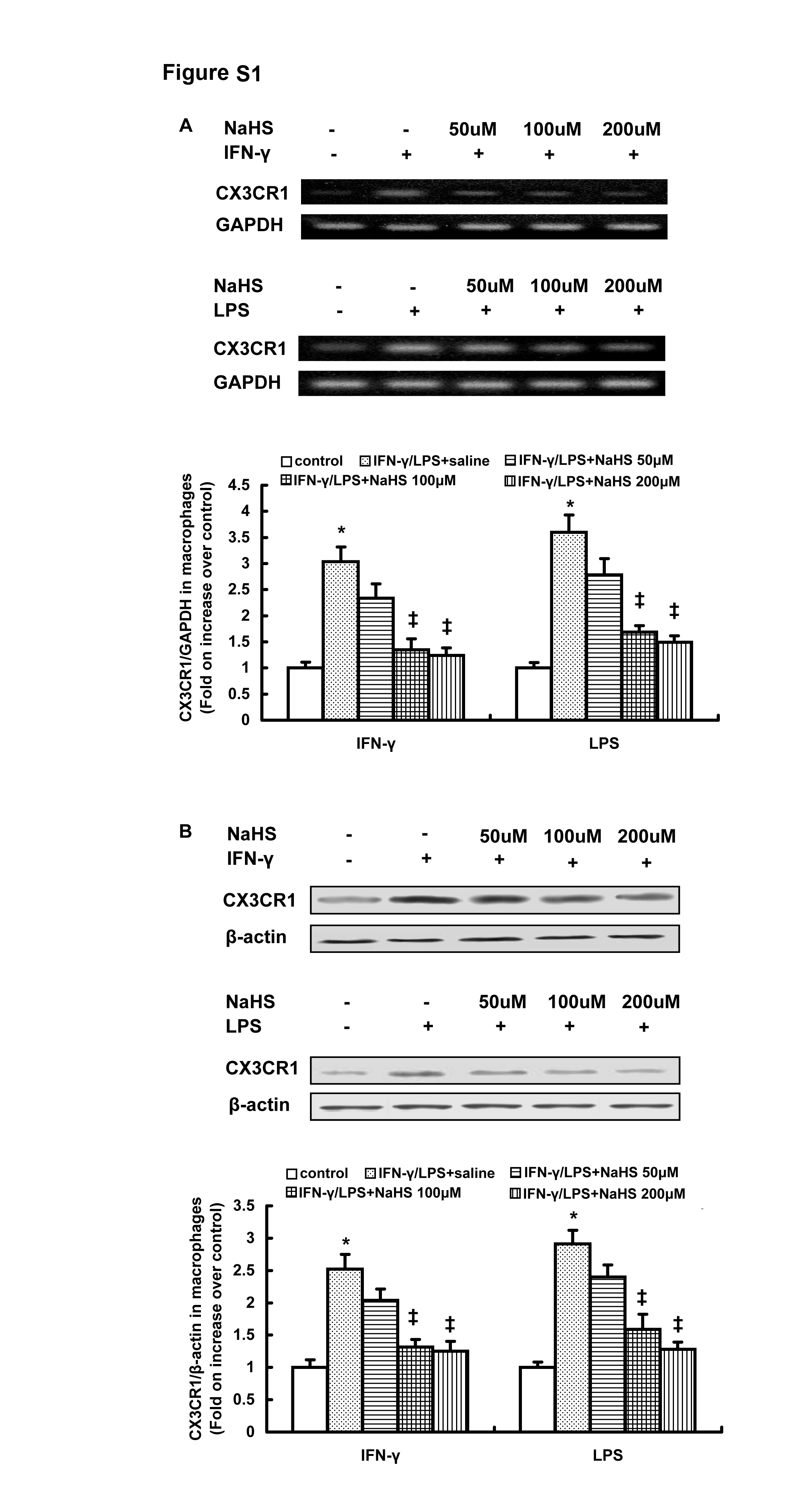

Supplement: Figure S1 — Effect of NaHS on CX3CR1 expression in mouse peritoneal macrophages stimulated with IFN-γ or LPS. RT-PCR analysis for CX3CR1 mRNA (A) and western blot analysis (B) for CX3CR1 protein expression were carried out as described in Materials and Methods. The data are means ± SEM of at least three independent experiments. *P<0.05, compared with unstimulated cells (control). ‡P<0.05, compared with stimulated cell pretreated with saline. (TIF) [file pone.0041147.s001.tif]

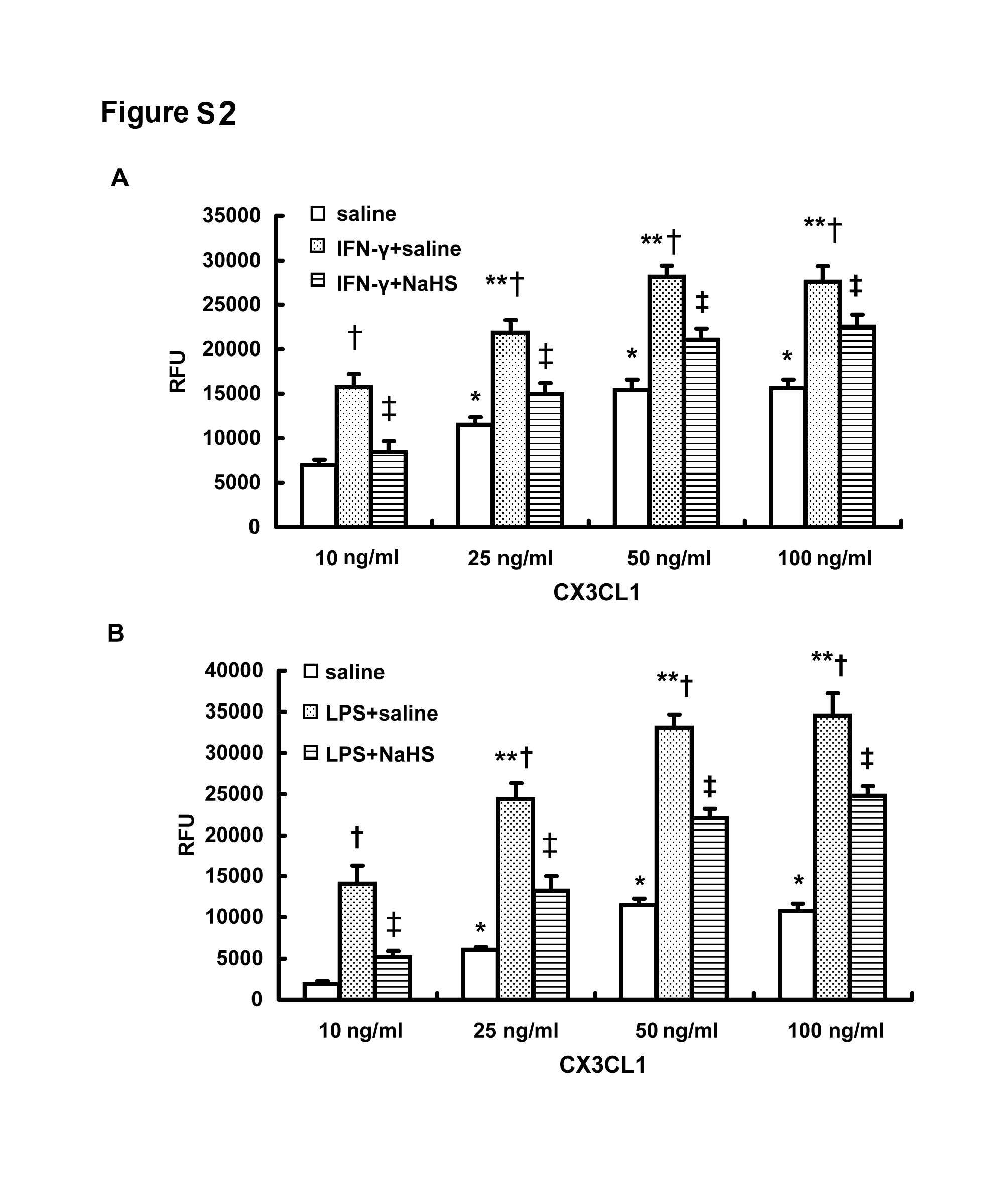

Supplement: Figure S2 — Effect of NaHS (100 µM) on chemotactic response to increasing doses of CX3CL1 in RAW264.7 cells stimulated with IFN-γ- or LPS. Chemotaxis of RAW264.7 cells towards CX3CL1 was assayed as described in Materials and Methods. The data are means ± SEM of at least three independent experiments. *P<0.05, compared with saline-pretreated, unstimulated cells (CX3CL1 10 ng/ml). **P<0.05, compared with saline-pretreated, stimulated cells (CX3CL1 10 ng/ml). †P<0.05, compared with saline-pretreated, unstimulated cells (CX3CL1 at the same concentration). ‡P<0.05, saline-pretreated, stimulated cells (CX3CL1 at the same concentration). (TIF) [file pone.0041147.s002.tif]

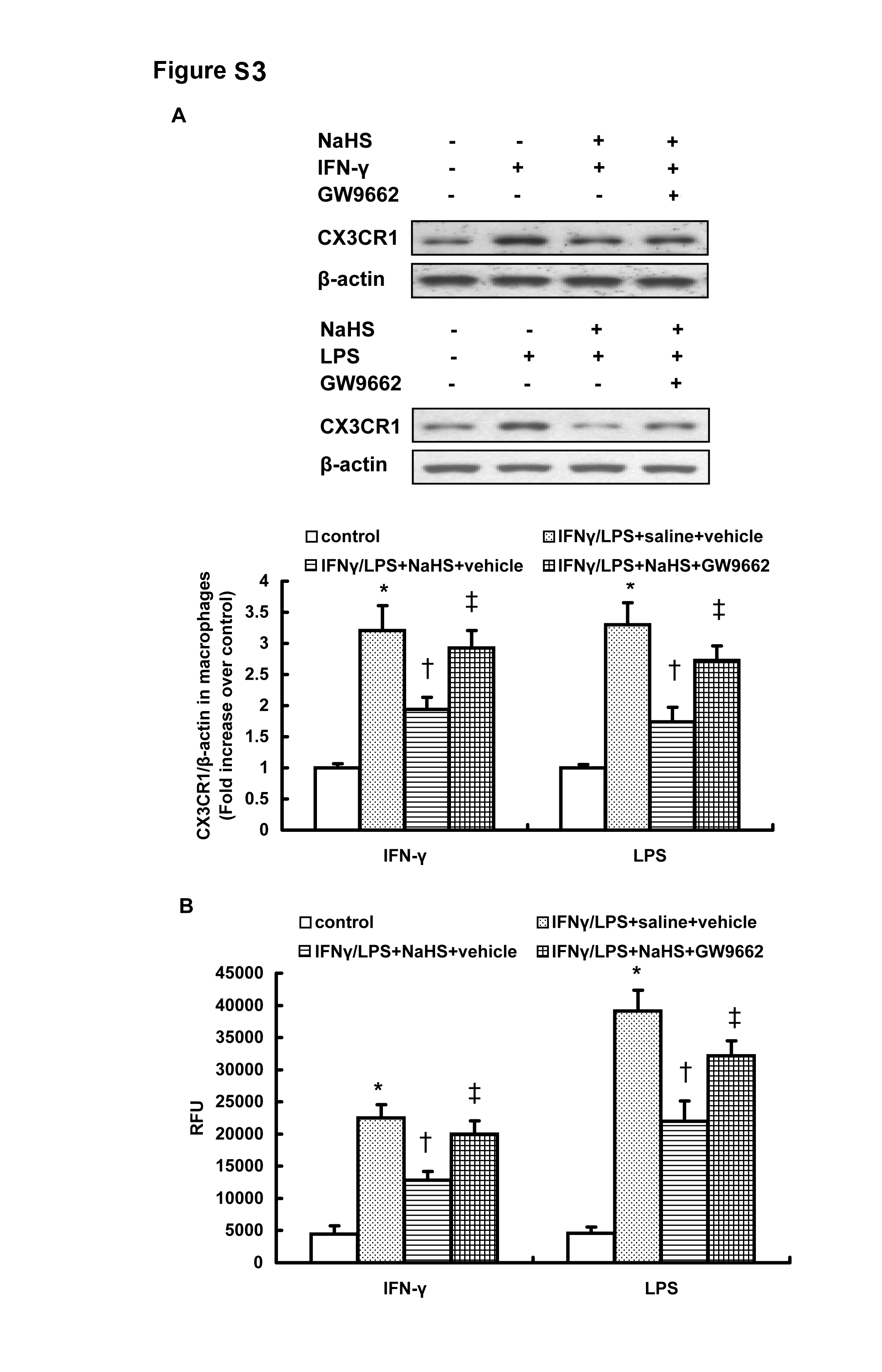

Supplement: Figure S3 — Effect of pretreatment with GW9662 on NaHS-induced downregulation of CX3CR1 expression and NaHS-induced inhibition of CX3CR1-mediated chemotaxis in IFN-γ or LPS-stimulated mouse peritoneal macrophages. Cells were incubated with GW9662 (10 µM) or vehicle for 1 hour, further incubated with NaHS (100 µM) or saline for 6 hours and then stimulated with IFN-γ (500 U/ml) or LPS (10 µg/ml) for 12 hours in the continuous presence of NaHS or saline. Western blot analysis for CX3CR1 expression (A) and chemotaxis towards CX3CL1 (50 ng/ml) (B) were assayed as described in Materials and Methods. The data are means ± SEM of at least three independent experiments. *P<0.05, compared with unstimulated cells (control). †P<0.05, compared with stimulated cells treated with saline and vehicle. ‡P<0.05, compared with stimulated cell treated with NaHS and vehicle. (TIF) [file pone.0041147.s003.tif]

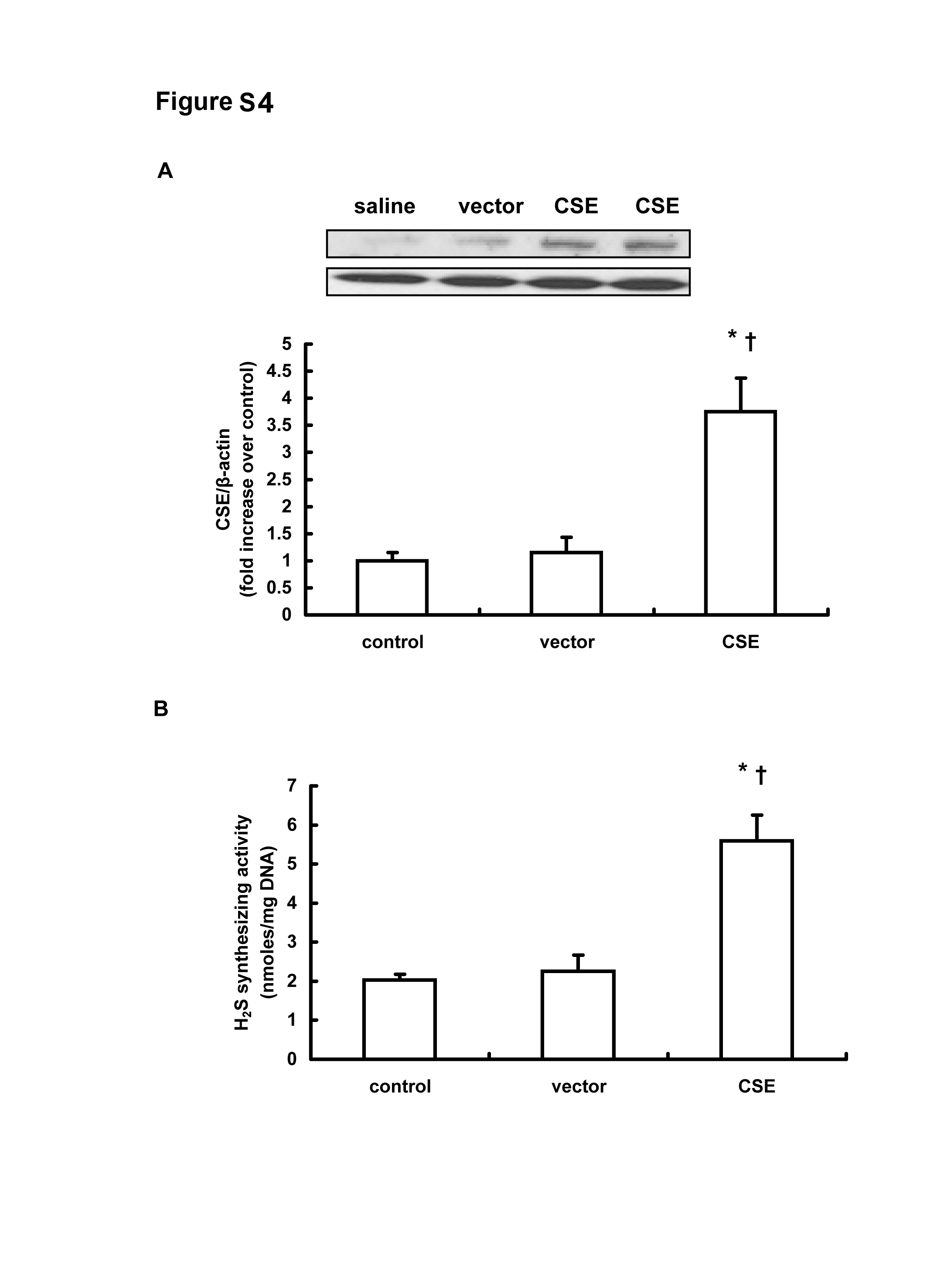

Supplement: Figure S4 — Transfection of CSE cDNA construct in RAW264.7 cell. Cells were transfected with CSE cDNA construct or an identical empty vector lacking a cDNA insert as a control. The expression of CSE was verified by western blot analysis (A) and the H2S synthesizing activity (B) was assayed as described in Materials and Methods. The data are means ± SEM of four independent experiments. *P<0.05, compared with control cells. †P<0.05, compared with cells transfected with empty vector. (TIF) [file pone.0041147.s004.tif]

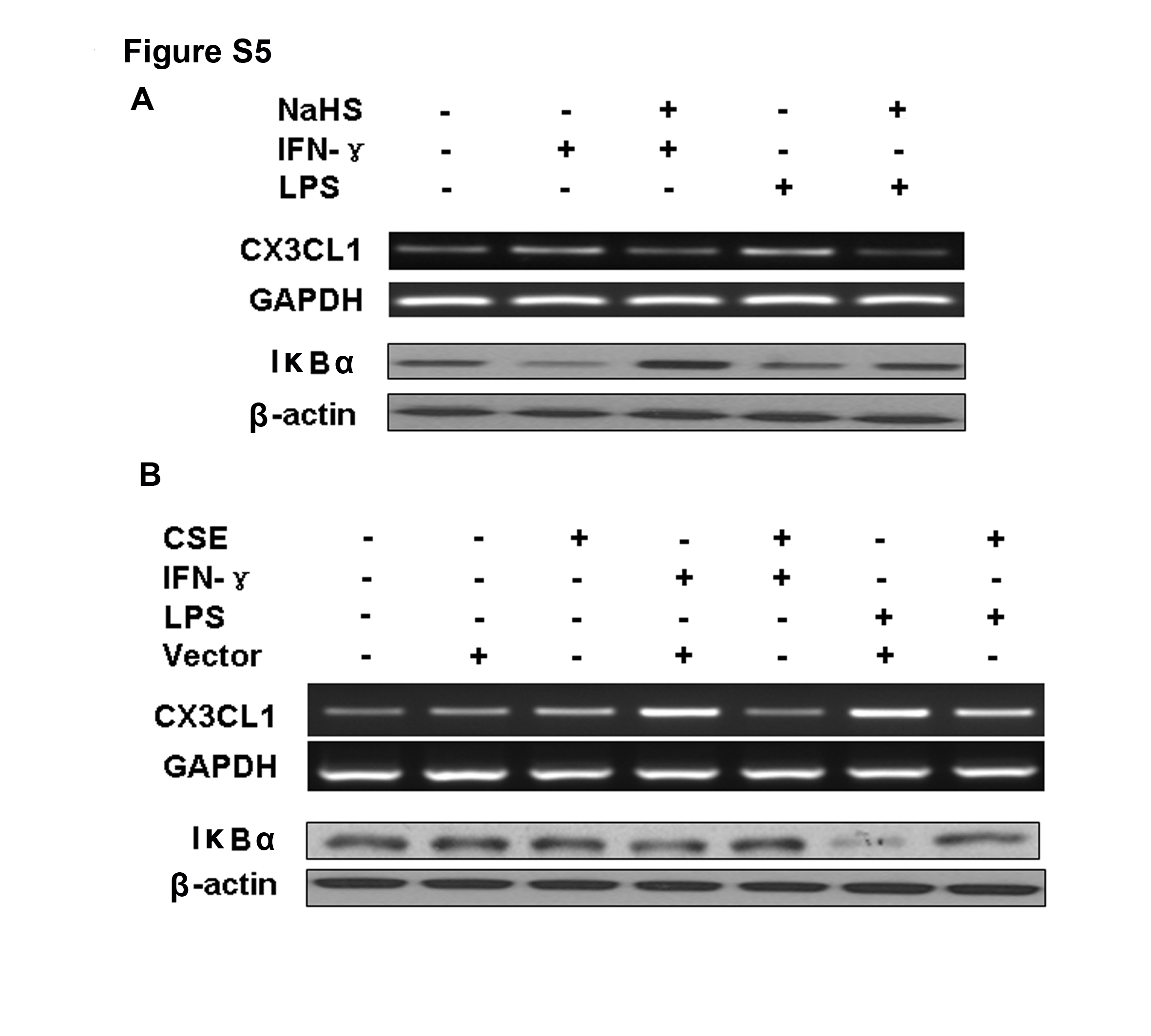

Supplement: Figure S5 — Effect of NaHS or CSE overexpression on CX3CL1 mRNA expression and IκBα content in macrophages stimulated with IFN-γ or LPS. (A) Mouse peritoneal macrophages were pre-incubated with saline or NaHS (100 µM) for 6 hours and then stimulated with IFN-γ or LPS for 12 hours in the continuous presence of NaHS or saline. (B) RAW264.7 cells were transfected with CSE cDNA construct or empty vector and then were stimulated with IFN-γ or LPS for 12 hours. RT-PCR analysis for CX3CL1 mRNA and western blot analysis for IκBα content were carried out as described in Materials and Methods. (TIF) [file pone.0041147.s005.tif]

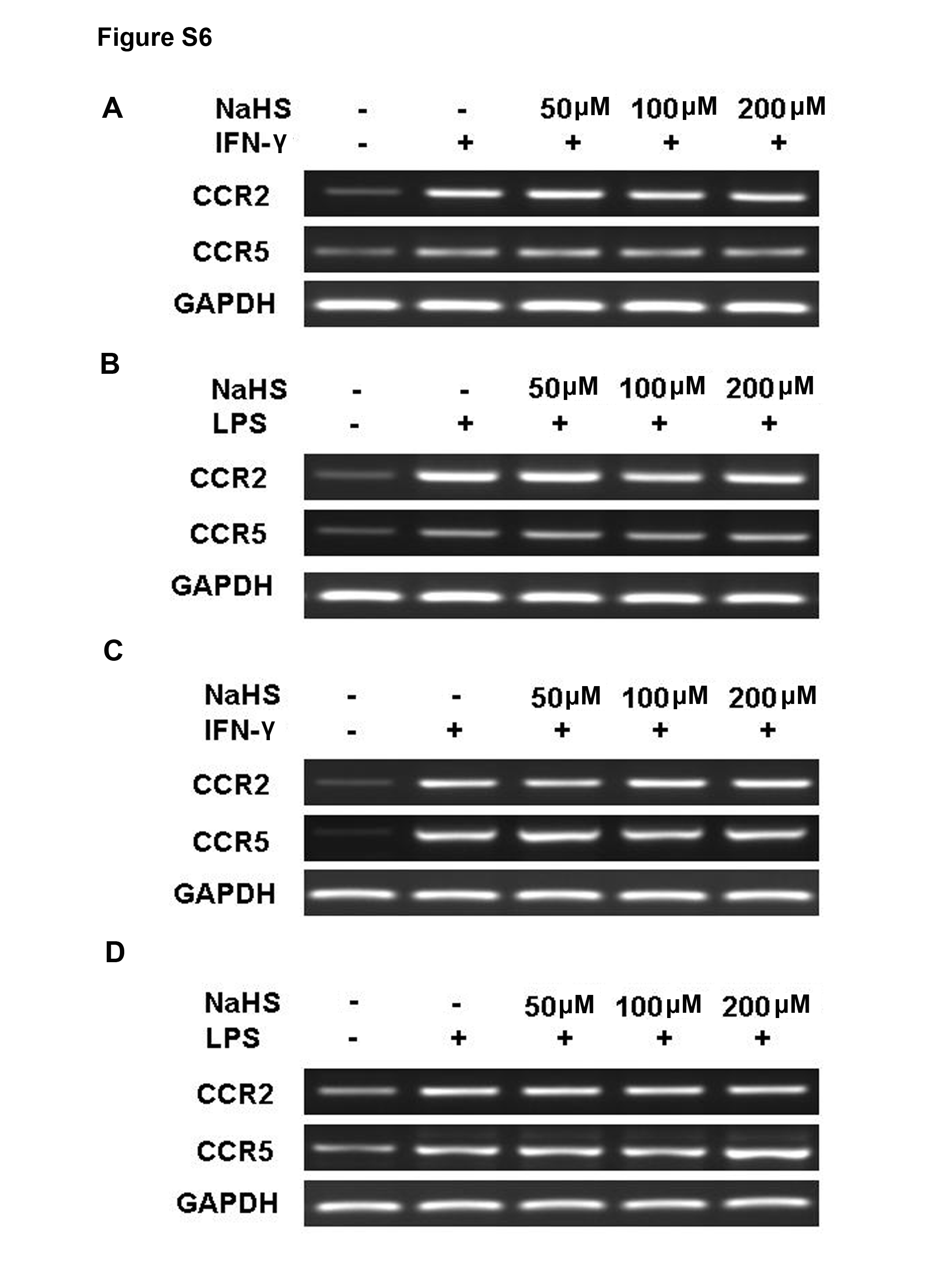

Supplement: Figure S6 — Effect of NaHS on CCR2 and CCR5 mRNA expression in macrophages stimulated with IFN-γ or LPS. RAW264.7 cells (A, B) or mouse peritoneal macrophages (C, D) were pre-incubated with saline or NaHS (50 µM, 100 µM, 200 µM) for 6 hours and then stimulated with IFN-γ or LPS for 12 hours in the continuous presence of NaHS or saline. RT-PCR analysis for CCR2 and CCR5 mRNA was carried out as described in Materials and Methods. (TIF) [file pone.0041147.s006.tif]

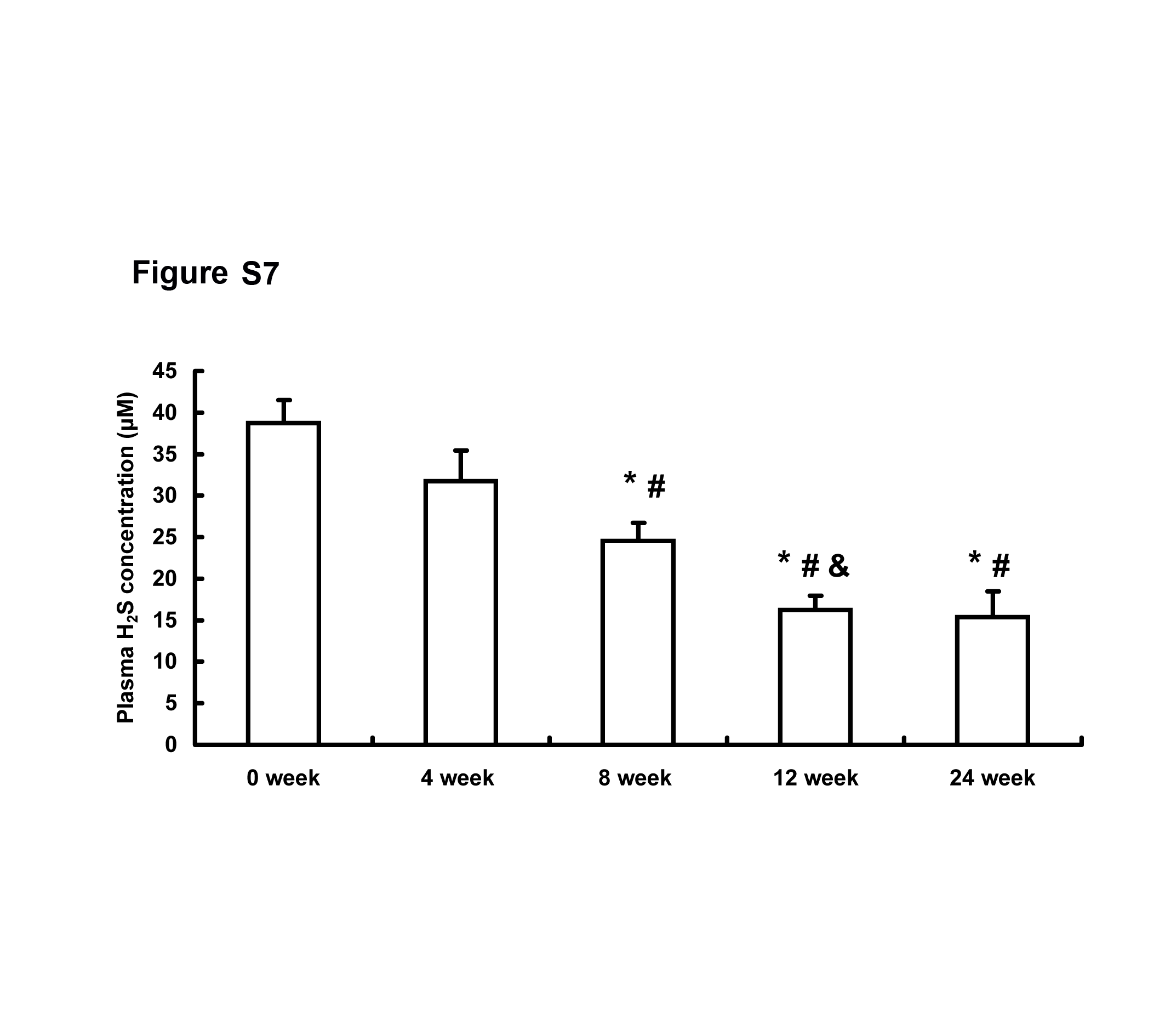

Supplement: Figure S7 — Alterations in plasma H2S level during the development of atherosclerosis in fat-fed apoE−/− mice. Plasma H2S levels were assayed at indicated time points (0, 4, 8, 12, 24 weeks after fat feeding) by sulfur-sensitive electrode method. Results shown are the mean ± SEM (n = 6 animals in each group). *P<0.05, compared with the basal level at 0 weeks. #P<0.05, compared with mice sacrificed 4 weeks after fat feeding. & P<0.05, compared with mice sacrificed 8 weeks after fat feeding. (TIF) [file pone.0041147.s007.tif]

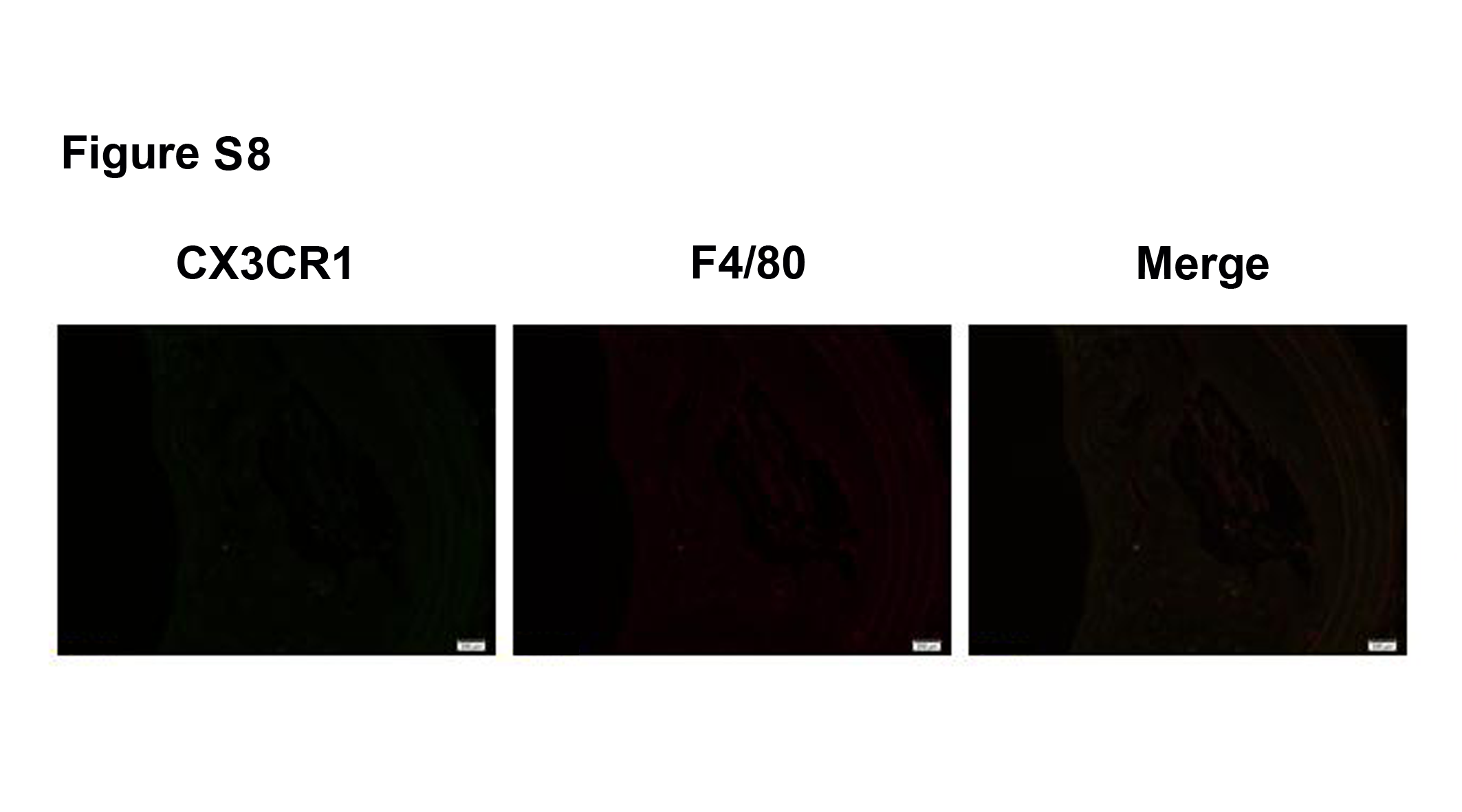

Supplement: Figure S8 — Negative control immunofluorescent staining in BCA with mouse IgG1 isotype control and Alexa Fluor 488 or Alexa Fluor 594 labeled secondary antibodies. Scale bar for histological images = 20 µm. (TIF) [file pone.0041147.s008.tif]

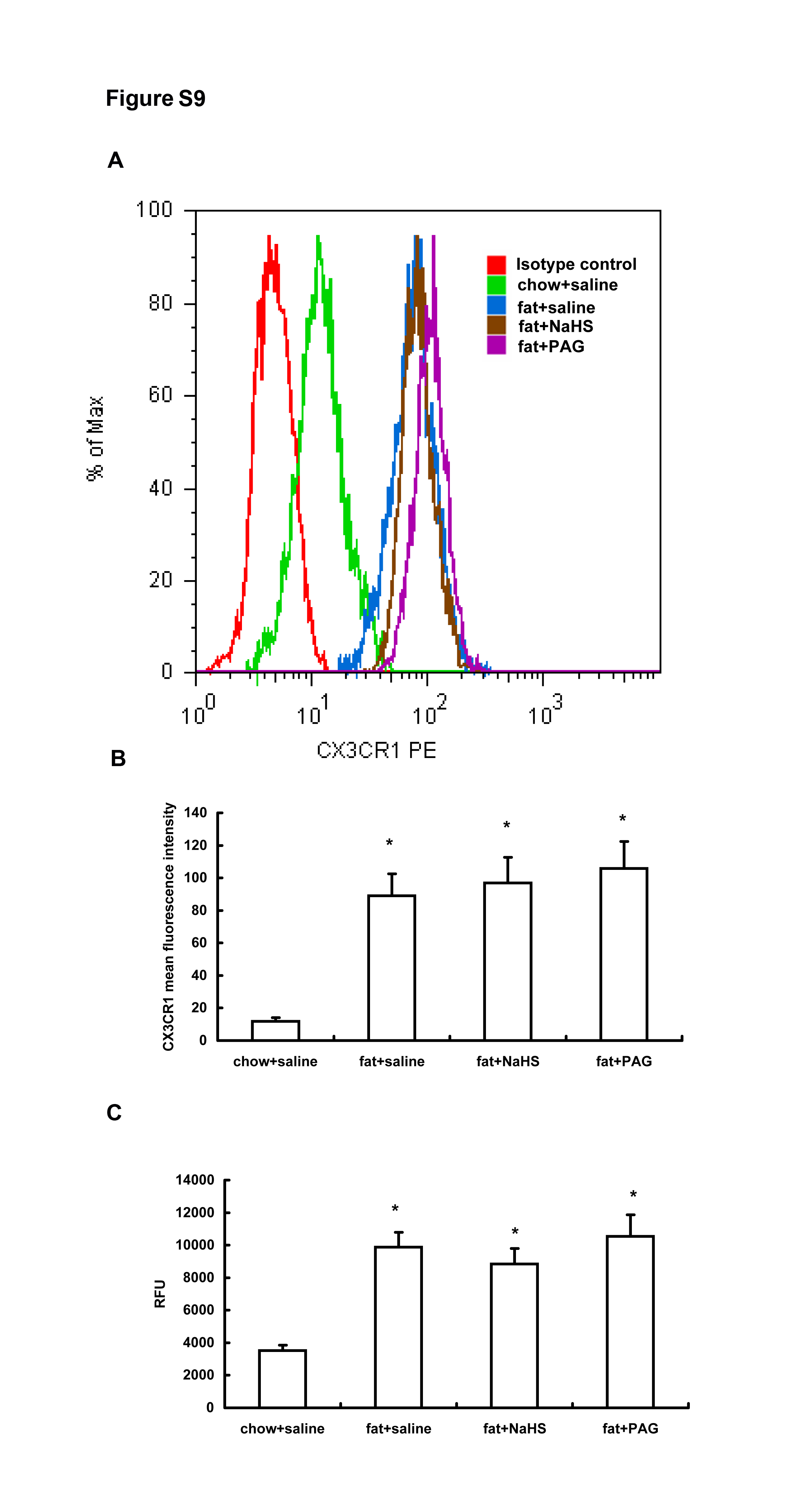

Supplement: Figure S9 — Effect of H2S on CX3CR1 expression and CX3CL1-mediated chemotaxis of circulating monocytes isolated from fat-fed apoE−/− mice. ApoE−/− mice (n = 10 in each group) were fed a high-fat diet and at the same time received saline, NaHS (1 mg/kg, daily, i.p.), or PAG (10 mg/kg, daily, i.p.) and sacrificed 8 weeks after fat feeding. Whole blood was collected and the mononuclear cell fraction was isolated, and then incubated with antibodies to detect CD11b+CX3CR1+ cell populations using flow cytometry (A). CX3CR1 mean fluorescence intensity (B) and CX3CL1-induced chemotaxis (C) were determined among treatment groups. Results are expressed as mean±SEM. *P<0.05, compared with chow+saline group. (TIF) [file pone.0041147.s009.tif]
