# Supplementary material for: Hydrogen Sulfide Inhibits the Development of Atherosclerosis with Suppressing CX3CR1 and CX3CL1 Expression
Source: PLoS One. 2012 Jul 18;7(7):e41147. doi: 10.1371/journal.pone.0041147 (PMC3399807; doi:10.1371/journal.pone.0041147)
Supplement: Table S1 — Effect of NaHS on CX3CL1 level in RAW264.7culture media stimulated with IFN-γ or LPS. (DOC) [file pone.0041147.s010.doc]

**Table S1** Effect of NaHS on CX3CL1 level in RAW264.7culture media stimulated with IFN-γ or LPS

|  |  |  |  | IFN-γ |  |  |  | LPS |  |
| --- | --- | --- | --- | --- | --- | --- | --- | --- | --- |
|  | control | IFN-γ+saline | NaHS | NaHS | NaHS | LPS+saline | NaHS | NaHS | NaHS |
|  |  |  | 50μM | 100μM | 200μM |  | 50μM | 100μM | 200μM |
| CX3CL1 | Undetectable | 12.57±1.81 | 10.06±1.01 | 7.03±0.81** | 6.18±0.51** | 18.17±1.28 | 14.40±1.34 | 10.51±1.16*# | 9.94±0.63*# |
| (ng/ml) |  |  |  |  |  |  |  |  |  |

** P<0.05, vs. IFN-γ+saline group; * P<0.05, vs. LPS+saline group; # P<0.05, vs. LPS+NaHS 50μM
