# Supplementary material for: Hydrogen Sulfide Inhibits the Development of Atherosclerosis with Suppressing CX3CR1 and CX3CL1 Expression
Source: PLoS One. 2012 Jul 18;7(7):e41147. doi: 10.1371/journal.pone.0041147 (PMC3399807; doi:10.1371/journal.pone.0041147)
Supplement: Table S2 — Effect of NaHS on CX3CL1 in stimulated mouse peritoneal macrophages. (DOC) [file pone.0041147.s011.doc]

**Table S2** Effect of NaHS on CX3CL1 in stimulated mouse peritoneal macrophages

|  | CX3CL1 mRNA | CX3CL1 (ng/ml)**/** | CX3CL1 in media | IκBα content | Nuclear NF-κB |
| --- | --- | --- | --- | --- | --- |
|  | (Fold increase over control group) | cell protein(mg/ml) | (ng/ml) | (Fold increase over control group) | activity |
| Control | 1.00±0.13 | 0.84±0.07 | 11.16±0.98 | 0.99±0.11 | 0.32±0.04 |
| IFN-γ+saline | 2.23±0.25* | 3.34±0.37* | 19.36±1.76* | 0.45±0.03* | 1.22±0.15* |
| IFN-γ+NaHS | 1.45±0.18** | 1.87±0.22** | 12.99±1.13** | 0.89±0.10** | 0.75±0.08** |
| LPS+saline | 3.78±0.43* | 10.4±0.091* | 29.87±2.41* | 0.24±0.02* | 1.92±0.20* |
| LPS+NaHS | 1.98±0.21# | 5.43±0.47# | 20.12±2.12# | 0.76±0.09# | 1.05±0.11# |

* P<0.05, vs. control group; ** P<0.05, vs. IFN-γ+saline; # P<0.05, vs. LPS+saline;
