# Supplementary material for: Hydrogen Sulfide Inhibits the Development of Atherosclerosis with Suppressing CX3CR1 and CX3CL1 Expression
Source: PLoS One. 2012 Jul 18;7(7):e41147. doi: 10.1371/journal.pone.0041147 (PMC3399807; doi:10.1371/journal.pone.0041147)
Supplement: Table S3 — Effect of CSE overexpression on CX3CL1 in stimulated RAW264.7 cells. (DOC) [file pone.0041147.s012.doc]

**Table S3** Effect of CSE overexpression on CX3CL1 in stimulated RAW264.7 cells

|  | CX3CL1 mRNA | CX3CL1 (ng/ml)**/** | CX3CL1 in media | IκBα content | Nuclear NF-κB |
| --- | --- | --- | --- | --- | --- |
|  | (Fold increase over control group ) | cell protein(mg/ml) | (ng/ml) | (Fold increase over control group ) | activity |
| Control | 1.00±0.11 | 0.35±0.05 | undetectable | 0.99±0.12 | 0.19±0.03 |
| saline+ vector | 1.25±0.16 | 0.76±0.08& | 10.16±1.10& | 0.82±0.10 | 0.68±0.09& |
| saline+CSE | 1.32±0.12 | 0.81±0.07& | 12.08±0.98& | 0.88±0.09 | 0.71±0.09& |
| IFN-γ+ vector | 3.15±0.36* | 4.65±0.50* | 21.12±1.43* | 0.41±0.05* | 1.76±0.12* |
| IFN-γ+ CSE | 1.77±0.14** | 2.11±0.18** | 13.56±1.02** | 0.74±0.09** | 1.02±0.17** |
| LPS+ vector | 3.45±0.40* | 13.45±1.28* | 26.44±2.96* | 0.31±0.05* | 2.34±0.27* |
| LPS+ CSE | 2.07±0.21# | 7.34±1.03# | 17.85±1.67# | 0.80±0.11# | 1.15±0.13# |

& P<0.05, vs. control group. * P<0.05, vs. saline+vector; ** P<0.05, vs. IFN-γ+ vector; # P<0.05, vs. LPS+ vector;
