# Supplementary material for: Hydrogen Sulfide Inhibits the Development of Atherosclerosis with Suppressing CX3CR1 and CX3CL1 Expression
Source: PLoS One. 2012 Jul 18;7(7):e41147. doi: 10.1371/journal.pone.0041147 (PMC3399807; doi:10.1371/journal.pone.0041147)
Supplement: Table S4 — Effect of H2S on CCL2 and CCL5 in stimulated RAW264.7. (DOC) [file pone.0041147.s013.doc]

**Table S4** Effect of H_2_S on CCL2 and CCL5 in stimulated RAW264.7

|  |  |  |  | IFN-γ |  |  |  | LPS |  |
| --- | --- | --- | --- | --- | --- | --- | --- | --- | --- |
|  | control | IFN-γ+saline | NaHS | NaHS | NaHS | LPS+saline | NaHS | NaHS | NaHS |
|  |  |  | 50μM | 100μM | 200μM |  | 50μM | 100μM | 200μM |
| CCL2 (pg/ml)**/** | 2.34±0.24 | 15.27±2.33* | 16.77±1.38 | 17.94±2.22 | 14.04±1.67 | 23.44±1.47* | 21.22±2.38 | 20.78±2.26 | 25.96±2.02 |
| cell protein (mg/ml) |  |  |  |  |  |  |  |  |  |
| CCL5 (pg/ml)**/** | 4.45±0.45 | 18.32±1.31* | 19.12±2.11 | 16.22±1.65 | 17.33±1.21 | 16.54±3.21* | 18.99±2.47 | 18.07±2.67 | 15.23±1.13 |
| cell protein (mg/ml) |  |  |  |  |  |  |  |  |  |

* P<0.05, vs. control group
