# Supplementary material for: Hydrogen Sulfide Inhibits the Development of Atherosclerosis with Suppressing CX3CR1 and CX3CL1 Expression
Source: PLoS One. 2012 Jul 18;7(7):e41147. doi: 10.1371/journal.pone.0041147 (PMC3399807; doi:10.1371/journal.pone.0041147)
Supplement: Table S5 — Effect of treatment with NaHS on blood pressure and plasma lipids. (DOC) [file pone.0041147.s014.doc]

**Table S5** Effect of treatment with NaHS on blood pressure and plasma lipids

|  | Weight | SBP | TC | TG | HDL-C | LDL-C |
| --- | --- | --- | --- | --- | --- | --- |
|  | (g) | (mmHg) | (mmol/L) | (mmol/L) | (mmol/L) | (mmol/L) |
| chow+saline | 32.95±3.36 | 112.82±11.25 | 8.43±2.90 | 1.20±0.40 | 1.03±0.39 | 6.85±3.01 |
| Fat + saline | 32.26±4.03 | 115.74±14.07 | 20.42±3.69* | 2.65±0.81* | 3.24±0.71* | 15.98±3.93* |
| Fat +early NaHS treatment | 30.26±4.31 | 102.36±10.82 | 19.89±4.69* | 2.81±1.30* | 3.30±0.81* | 16.82±3.38* |
| Fat +delayed NaHS treatment | 31.06±4.45 | 106.23±11.26 | 21.40±3.61* | 2.57±0.71* | 3.48±0.74* | 15.24±4.47* |

* P<0.05, vs chow + saline group; SBP: Systolic Blood Pressure; TC: Total Cholesterol; TG: Triglycerides; HDL-C: High-density Lipoprotein; LDL-C: Low-density Lipoprotein
