# Supplementary material for: Hydrogen Sulfide Inhibits the Development of Atherosclerosis with Suppressing CX3CR1 and CX3CL1 Expression
Source: PLoS One. 2012 Jul 18;7(7):e41147. doi: 10.1371/journal.pone.0041147 (PMC3399807; doi:10.1371/journal.pone.0041147)
Supplement: Table S6 — Effect of treatment with PAG on blood pressure and plasma lipids. (DOC) [file pone.0041147.s015.doc]

**Table S6** Effect of treatment with PAG on blood pressure and plasma lipids

|  | Weight | SBP | TC | TG | HDL-C | LDL-C |
| --- | --- | --- | --- | --- | --- | --- |
|  | (g) | (mmHg) | (mmol/L) | (mmol/L) | (mmol/L) | (mmol/L) |
| Fat + saline | 33.46±4.53 | 110.22±16.17 | 16.22±4.09 | 2.23±0.81 | 2.87±0.56 | 13.06±4.23 |
| Fat +PAG | 32.76±5.61 | 119.06±13.22 | 15.89±5.01 | 2.05±2.16 | 3.05±0.90 | 14.82±5.08 |
