# Supplementary material for: Hydrogen Sulfide Inhibits the Development of Atherosclerosis with Suppressing CX3CR1 and CX3CL1 Expression
Source: PLoS One. 2012 Jul 18;7(7):e41147. doi: 10.1371/journal.pone.0041147 (PMC3399807; doi:10.1371/journal.pone.0041147)
Supplement: Table S7 — Effect of H2S on CCL2, CCL5, CCR2 and CCR5 in vivo. (DOC) [file pone.0041147.s016.doc]

**Table S7** Effect of H_2_S on CCL2, CCL5, CCR2 and CCR5 in vivo

|  | Plasma | |  | mRNA level in aorta | |
| --- | --- | --- | --- | --- | --- |
|  | CCL2 (pg/ml) | CCL5(pg/ml) |  | CCR2 | CCR5 |
| chow+saline | 10.34±1.33 | 8.24±1.05 |  | 0.44±0.08 | 0. 25±0.04 |
| Fat + saline | 23.44±2.78* | 16.34±2.54* |  | 0.89±0.11* | 0.48±0.02* |
| Fat +early NaHS treatment | 26.79±3.54 | 17.56±3.87 |  | 1.02±0.17 | 0.38±0.04 |
| Fat +delayed NaHS treatment | 24.99±3.06 | 15.05±3.66 |  | 0.97±0.10 | 0.41±0.03 |

* P<0.05, vs. chow+saline group
