# Supplementary material for: Hydrogen Sulfide Inhibits the Development of Atherosclerosis with Suppressing CX3CR1 and CX3CL1 Expression
Source: PLoS One. 2012 Jul 18;7(7):e41147. doi: 10.1371/journal.pone.0041147 (PMC3399807; doi:10.1371/journal.pone.0041147)
Supplement: Table S8 — Effect of PAG on CCL2, CCL5, CCR2 and CCR5 in vivo. (DOC) [file pone.0041147.s017.doc]

**Table S8** Effect of PAG on CCL2, CCL5, CCR2 and CCR5 in vivo

|  | Plasma | |  | mRNA level in aorta | |
| --- | --- | --- | --- | --- | --- |
|  | CCL2 (pg/ml) | CCL5(pg/ml) |  | CCR2 | CCR5 |
| Fat + saline | 17.42±2.18 | 11.37±1.74 |  | 0.37±0.05 | 0.23±0.02 |
| Fat +PAG | 19.75±2.46 | 13.02±1.62 |  | 0.41±0.07 | 0.28±0.03 |
